# Supplementary figures and images for: Dbi1 is an oxidoreductase and an assembly chaperone for mitochondrial inner membrane proteins
Source: EMBO Rep. 2025 Jan 3;26(4):911–28. doi: 10.1038/s44319-024-00349-6 (PMC11850723; doi:10.1038/s44319-024-00349-6)

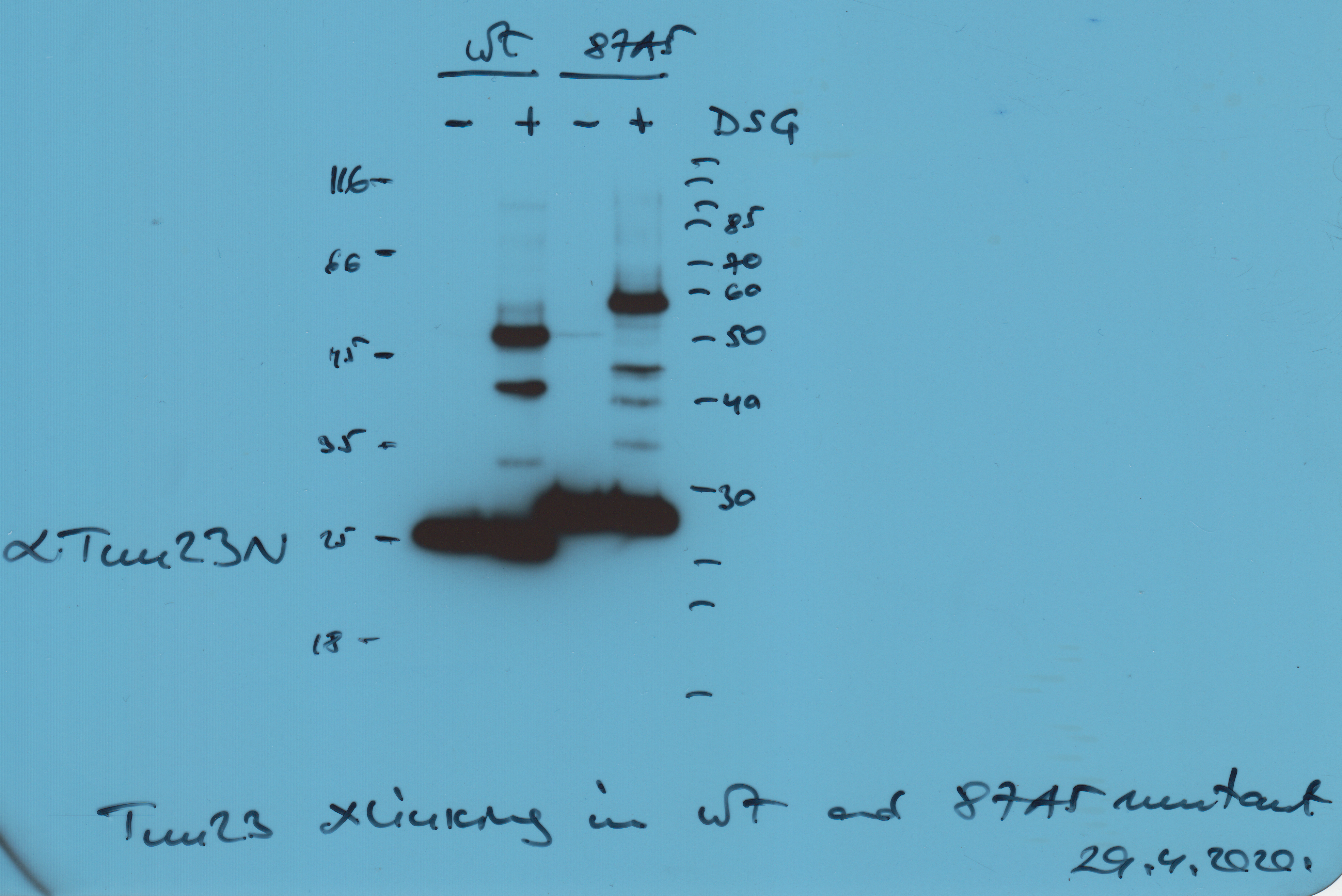

Supplement: Supplementary file 2 — Source data Fig. 1 [file 44319_2024_349_MOESM2_ESM.zip › Fig 1/1A/X link 87A5.tiff]

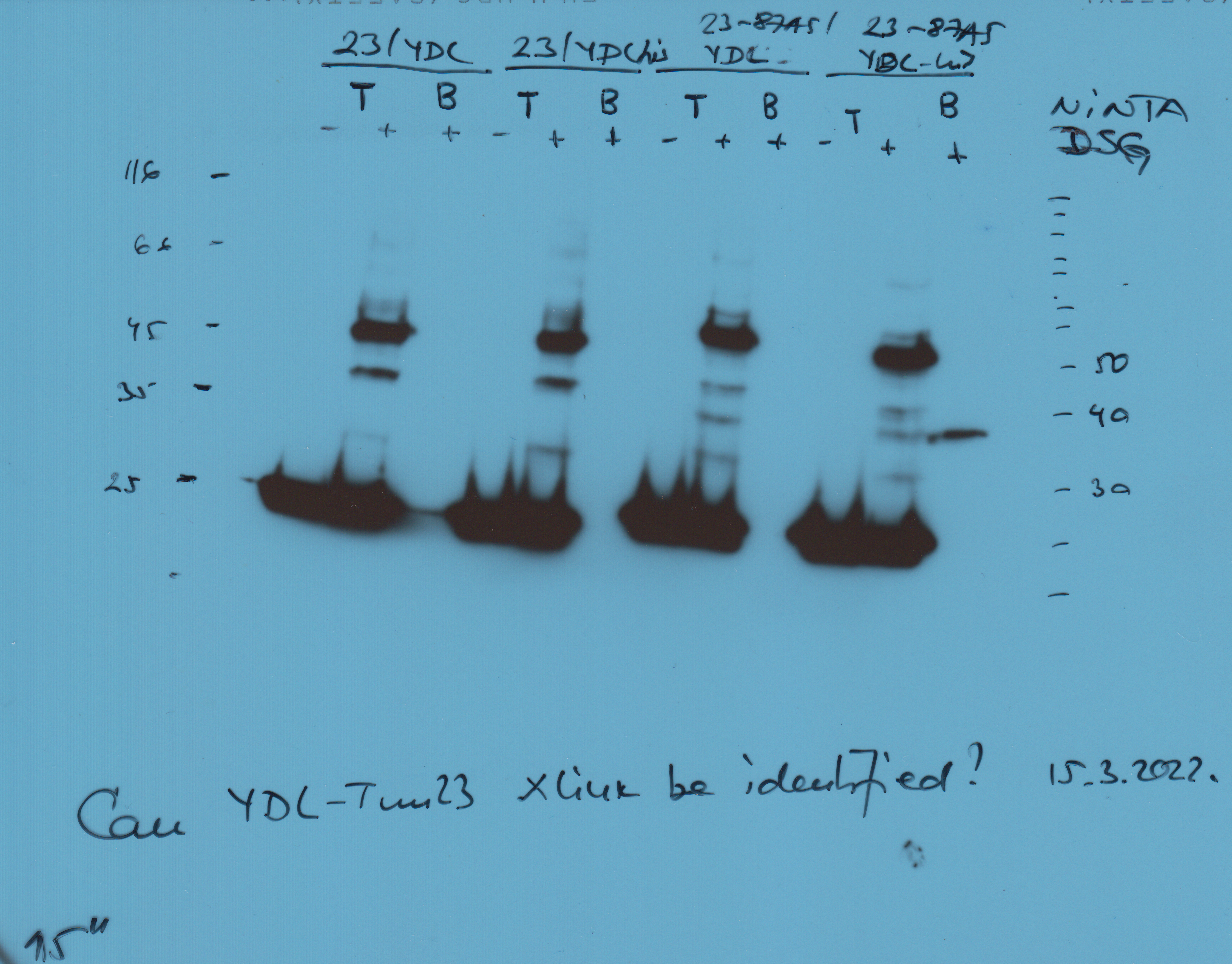

Supplement: Supplementary file 2 — Source data Fig. 1 [file 44319_2024_349_MOESM2_ESM.zip › Fig 1/1B/X link followed by pulldown.tiff]

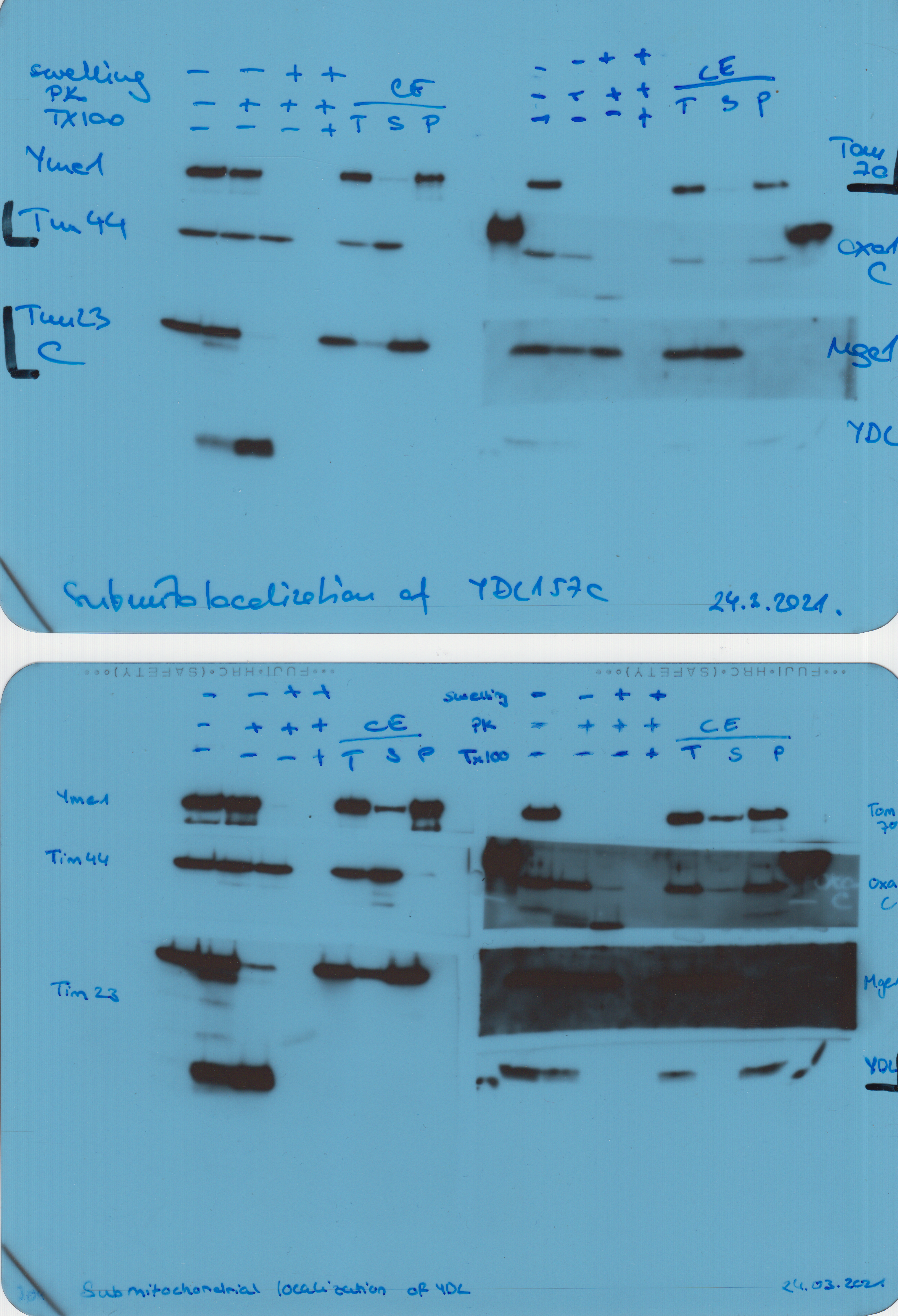

Supplement: Supplementary file 2 — Source data Fig. 1 [file 44319_2024_349_MOESM2_ESM.zip › Fig 1/1D/Submito localisation.tiff]

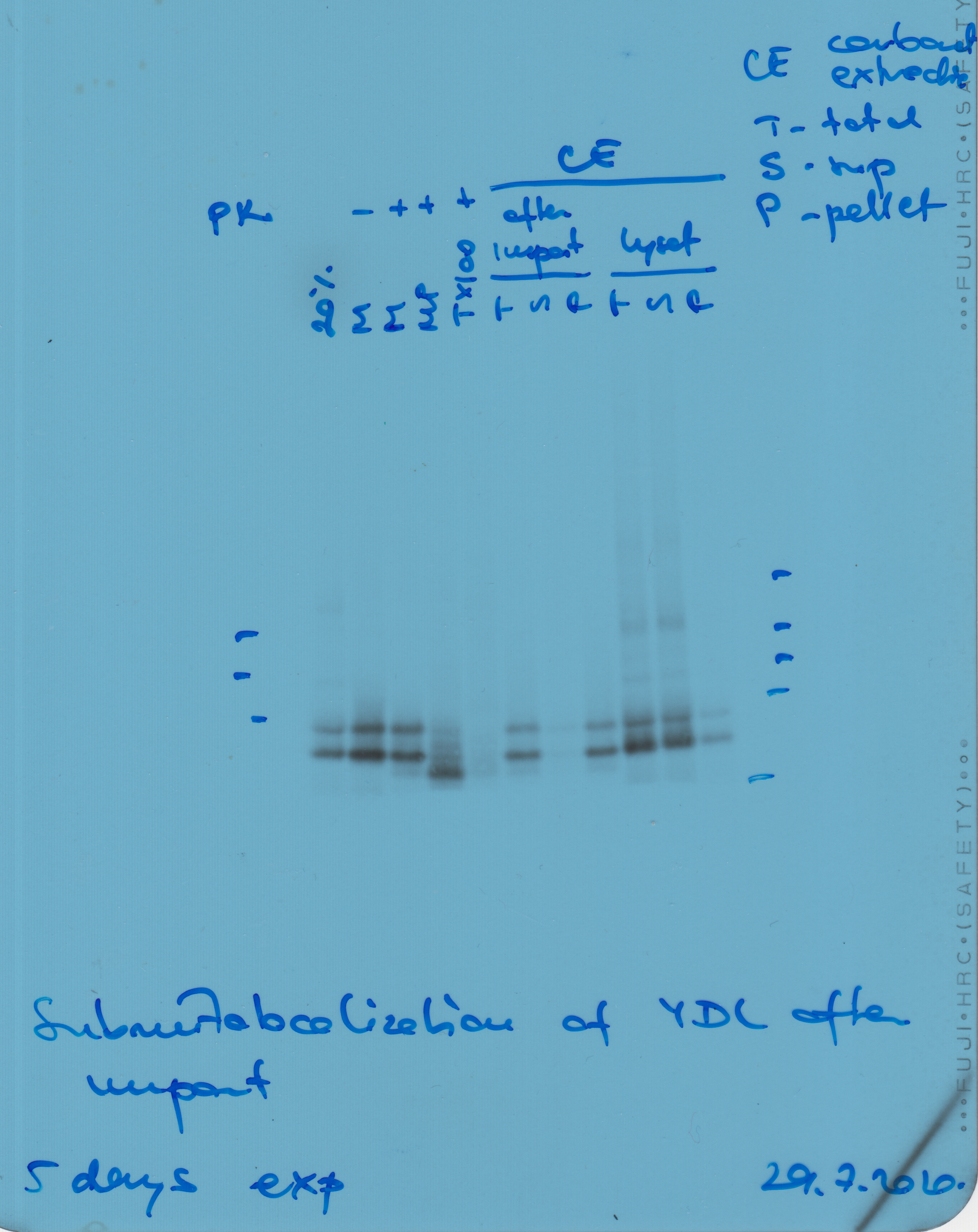

Supplement: Supplementary file 2 — Source data Fig. 1 [file 44319_2024_349_MOESM2_ESM.zip › Fig 1/1E/Submito localisation after import.tiff]

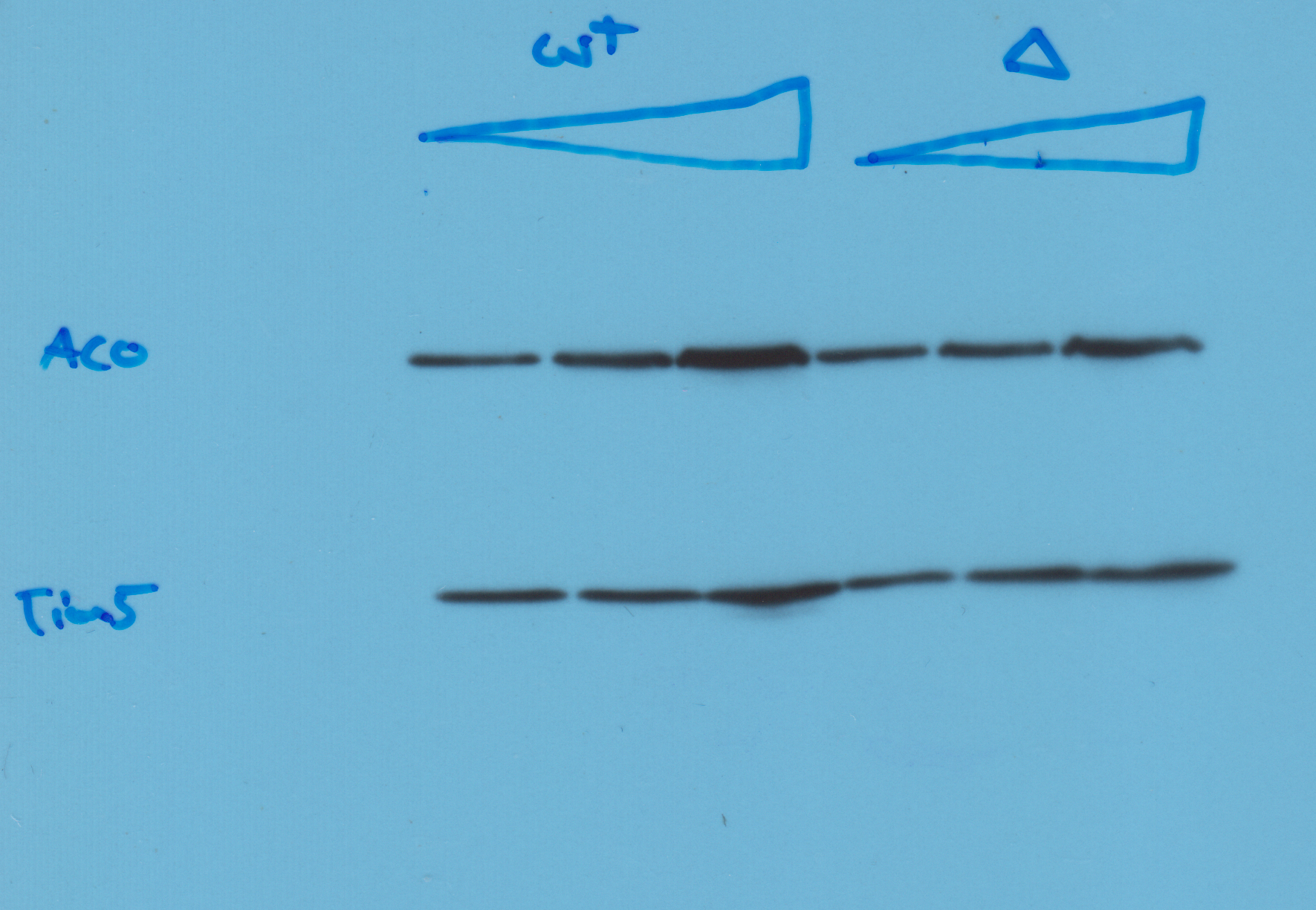

Supplement: Supplementary file 2 — Source data Fig. 1 [file 44319_2024_349_MOESM2_ESM.zip › Fig 1/1G/Mitoprofile Aco Tim50.tiff]

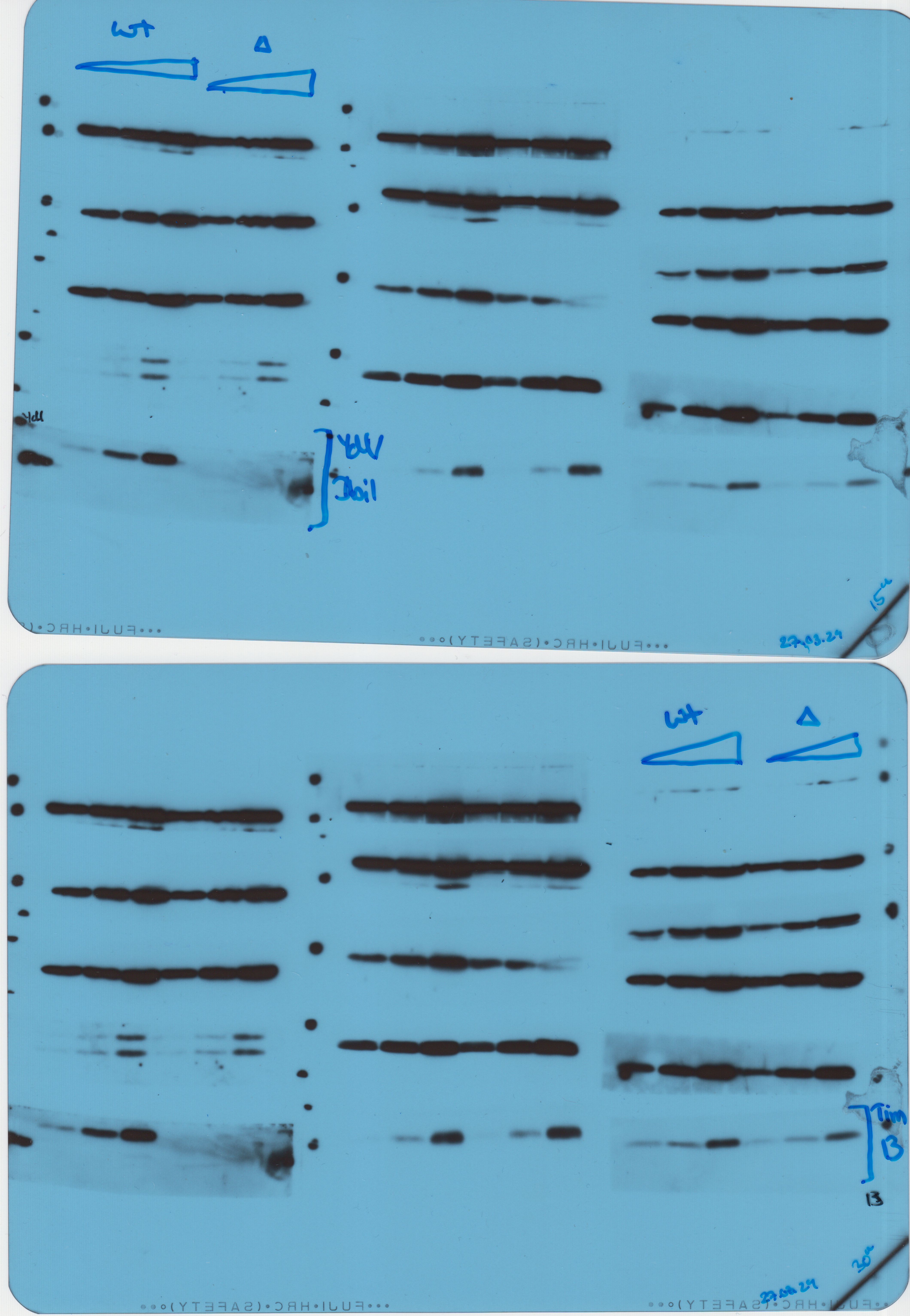

Supplement: Supplementary file 2 — Source data Fig. 1 [file 44319_2024_349_MOESM2_ESM.zip › Fig 1/1G/Mitoprofile Dbi1 Tim13.tiff]

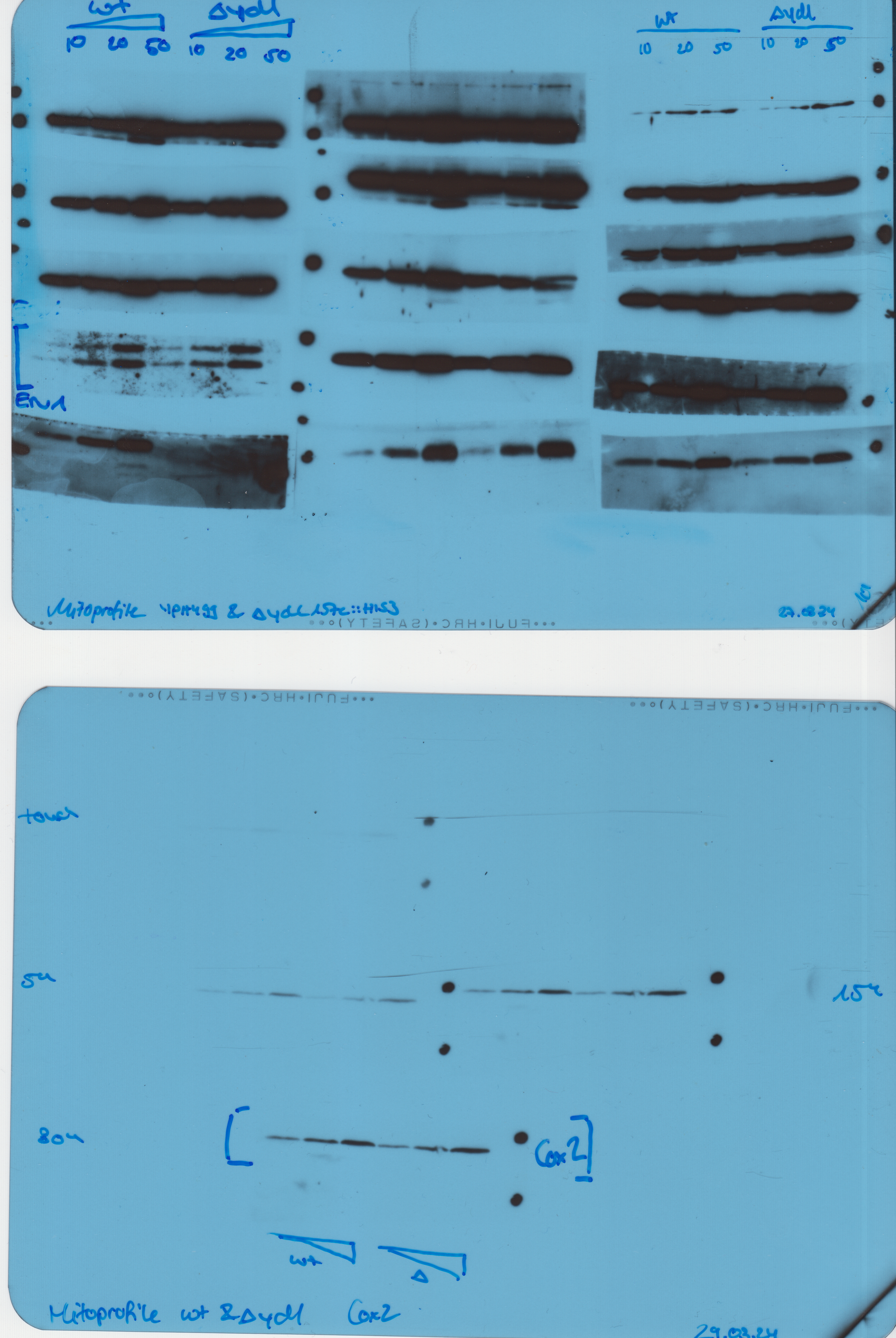

Supplement: Supplementary file 2 — Source data Fig. 1 [file 44319_2024_349_MOESM2_ESM.zip › Fig 1/1G/Mitoprofile Erv1 Cox2.tiff]

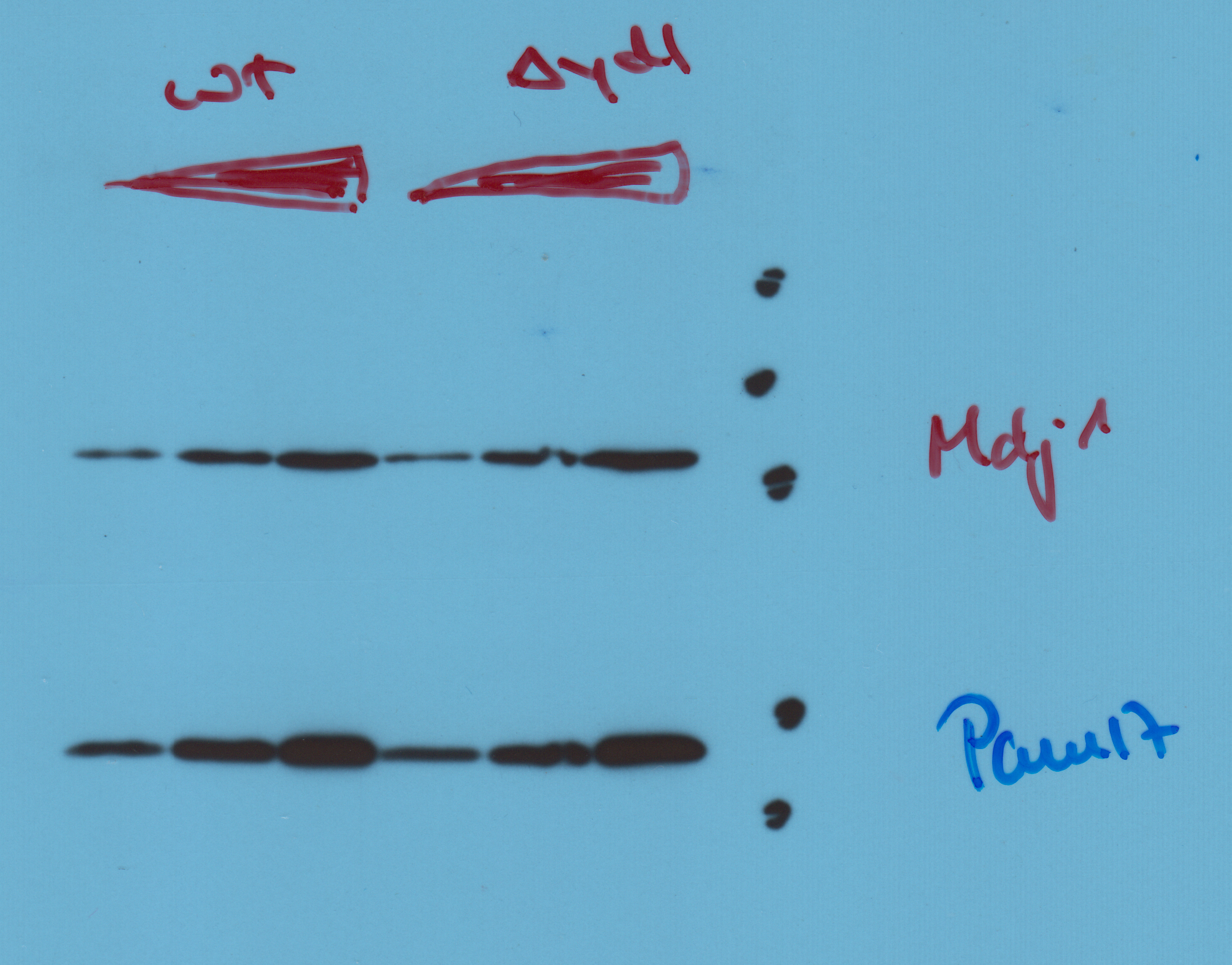

Supplement: Supplementary file 2 — Source data Fig. 1 [file 44319_2024_349_MOESM2_ESM.zip › Fig 1/1G/Mitoprofile Mdj1 Pam17.tiff]

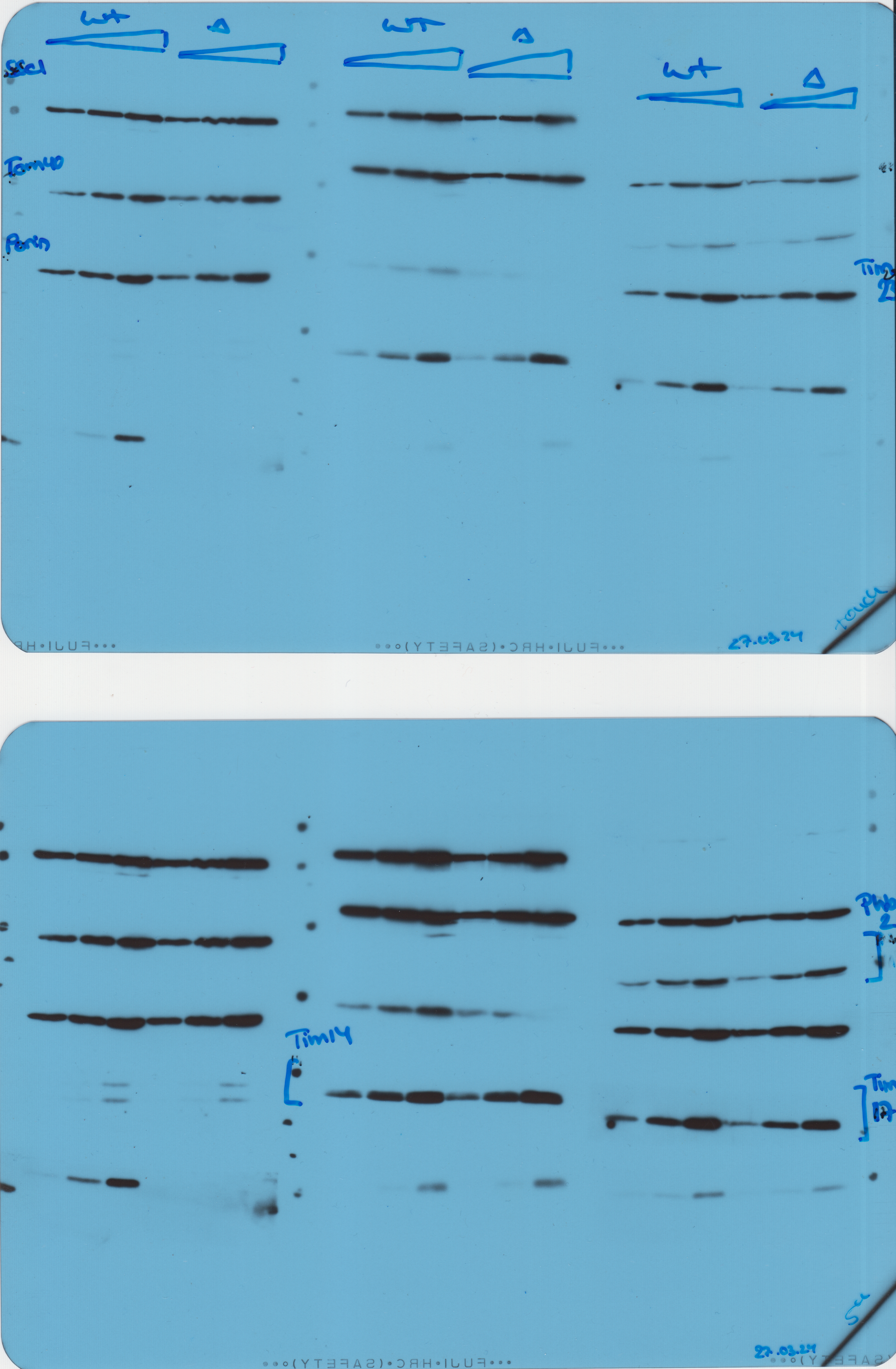

Supplement: Supplementary file 2 — Source data Fig. 1 [file 44319_2024_349_MOESM2_ESM.zip › Fig 1/1G/Porin Tom40 Ssc1 Tim23 Tim14 Tim17 Phb2 new.tiff]

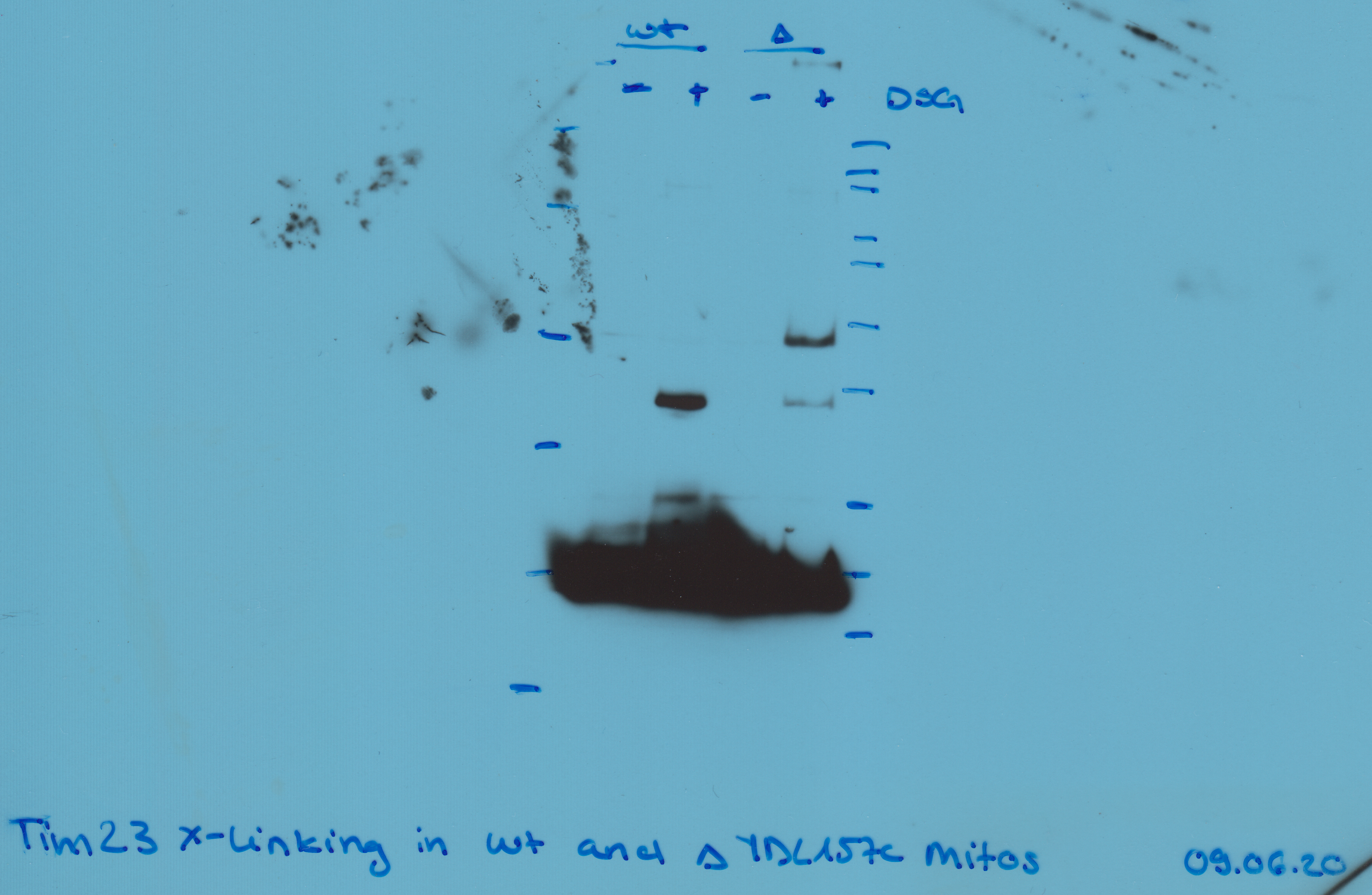

Supplement: Supplementary file 2 — Source data Fig. 1 [file 44319_2024_349_MOESM2_ESM.zip › Fig 1/1H/X link wt delta.tiff]

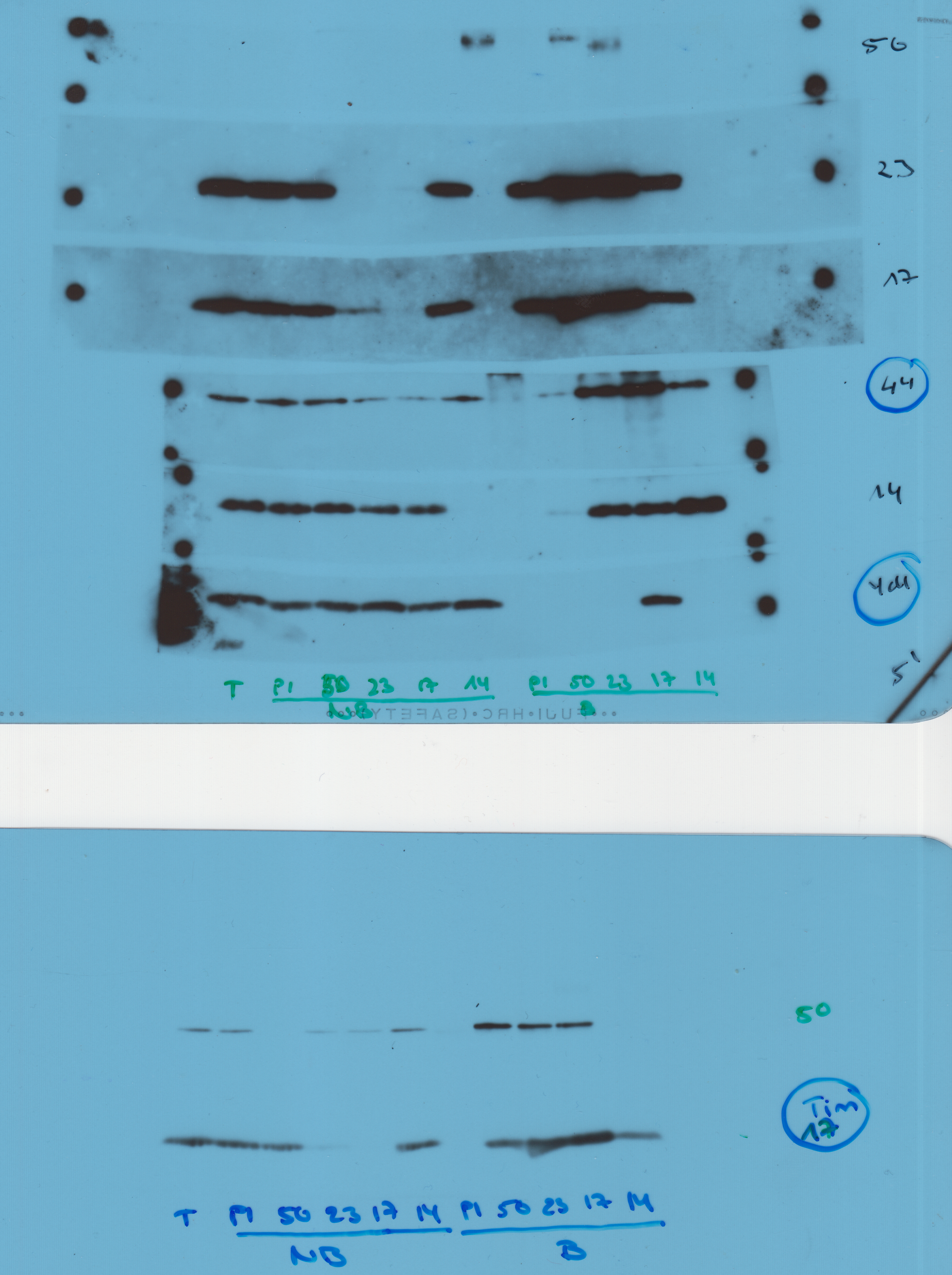

Supplement: Supplementary file 3 — Source data Fig. 2 [file 44319_2024_349_MOESM3_ESM.zip › Fig 2/2A/Co-IP wt Dbi1 Tim44 Tim17.tiff]

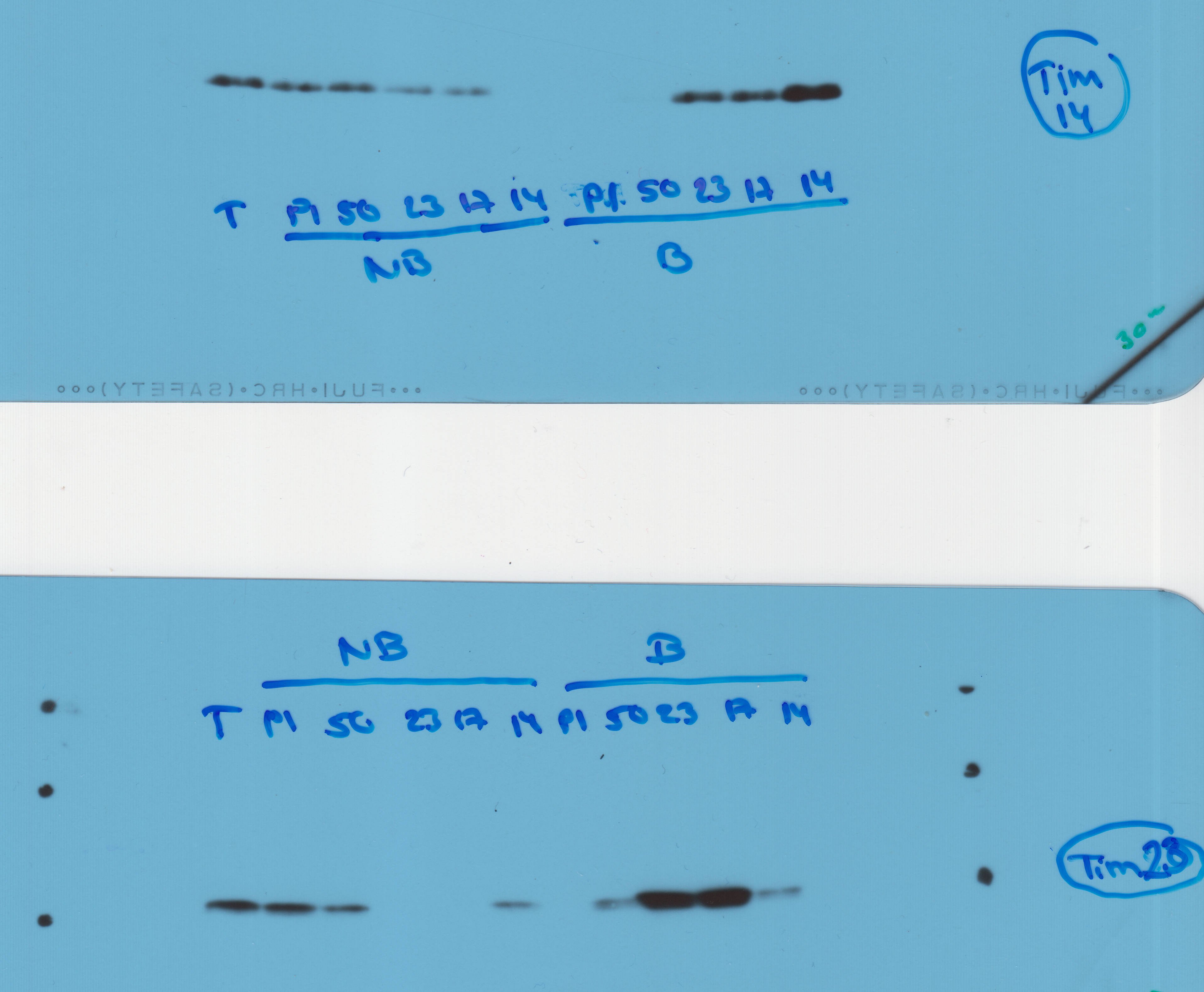

Supplement: Supplementary file 3 — Source data Fig. 2 [file 44319_2024_349_MOESM3_ESM.zip › Fig 2/2A/Co-IP wt Tim14 Tim23.tiff]

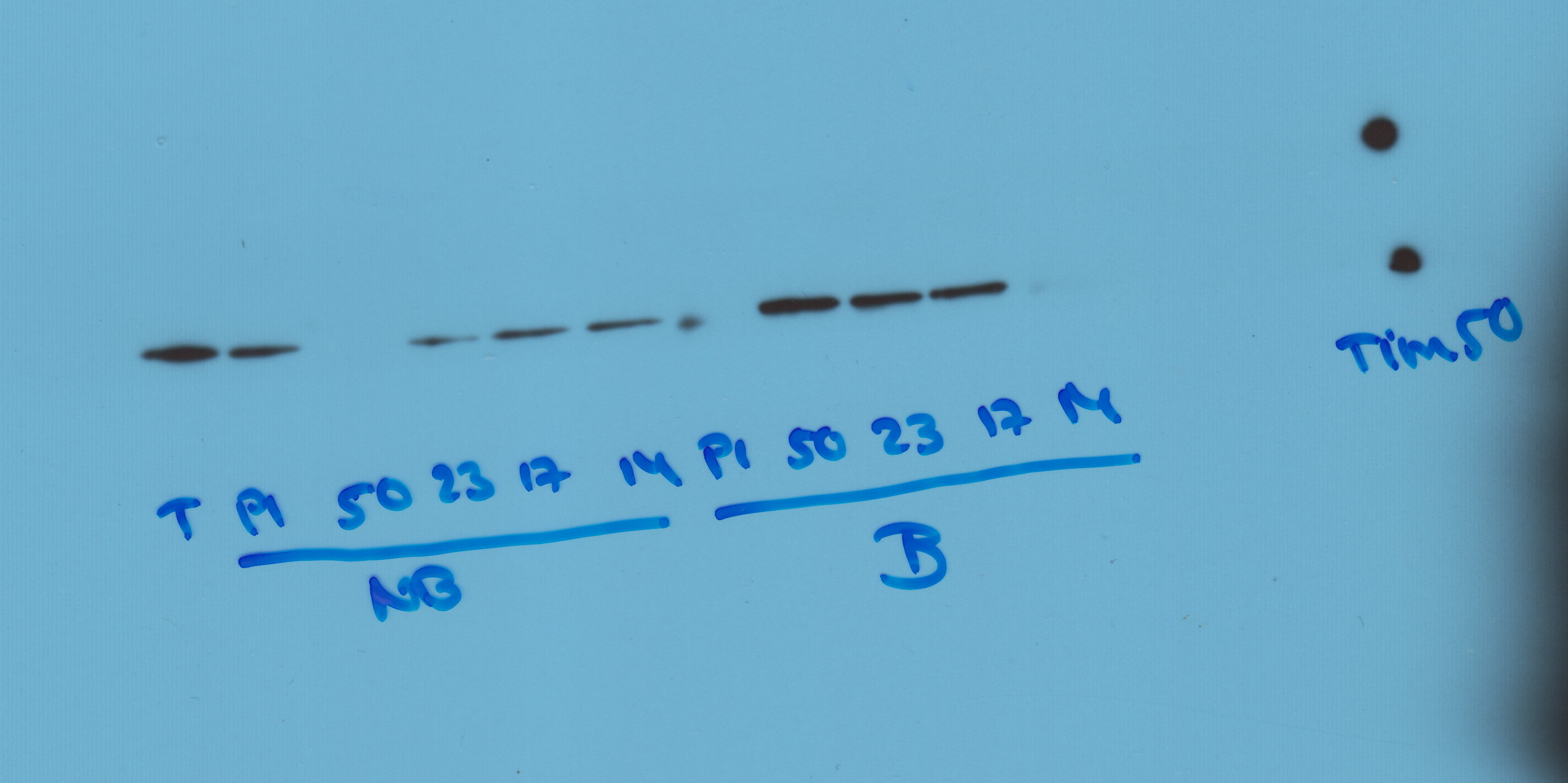

Supplement: Supplementary file 3 — Source data Fig. 2 [file 44319_2024_349_MOESM3_ESM.zip › Fig 2/2A/Co-IP wt Tim50.tiff]

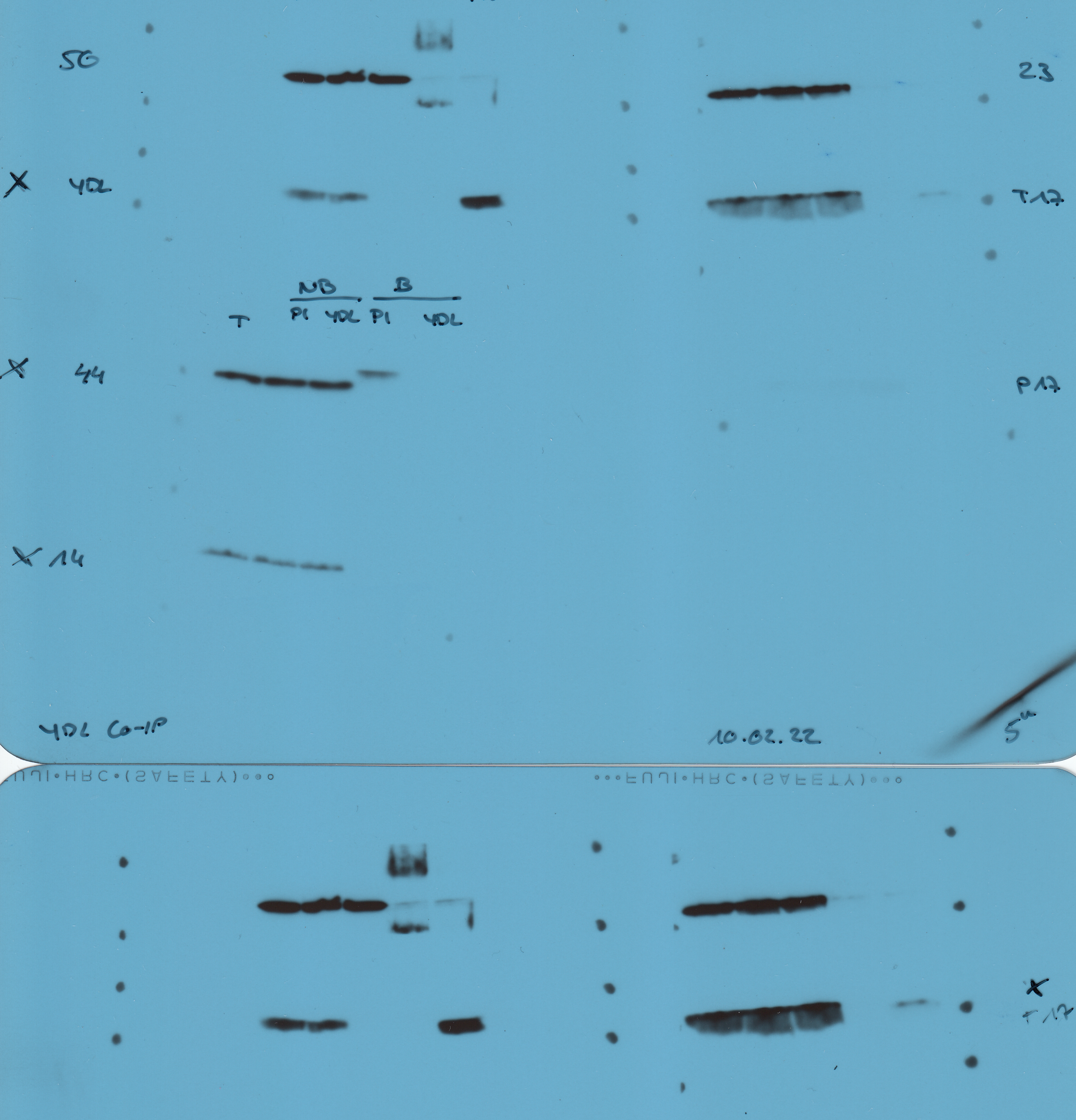

Supplement: Supplementary file 3 — Source data Fig. 2 [file 44319_2024_349_MOESM3_ESM.zip › Fig 2/2B/Co-IP dbi1 Dbi1, Tim44, Tim17, Tim14.tif]

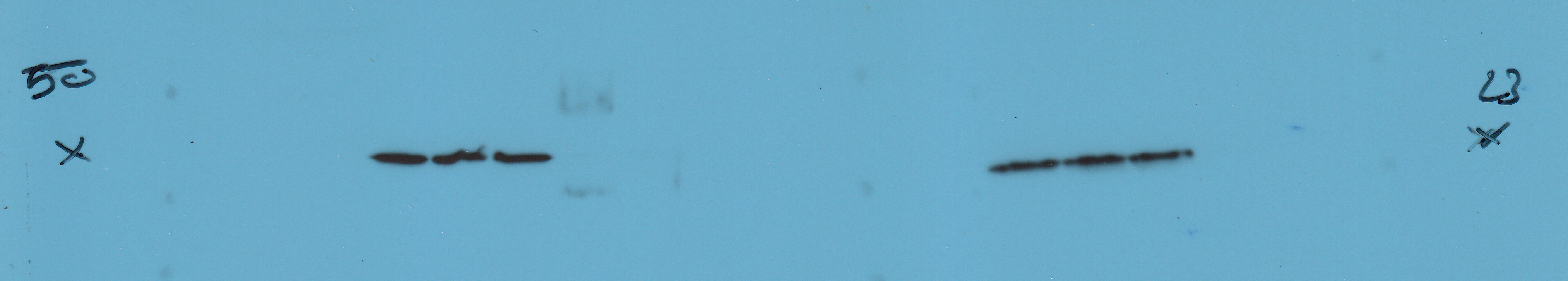

Supplement: Supplementary file 3 — Source data Fig. 2 [file 44319_2024_349_MOESM3_ESM.zip › Fig 2/2B/Co-IP dbi1 Tim50 Tim23.tif]

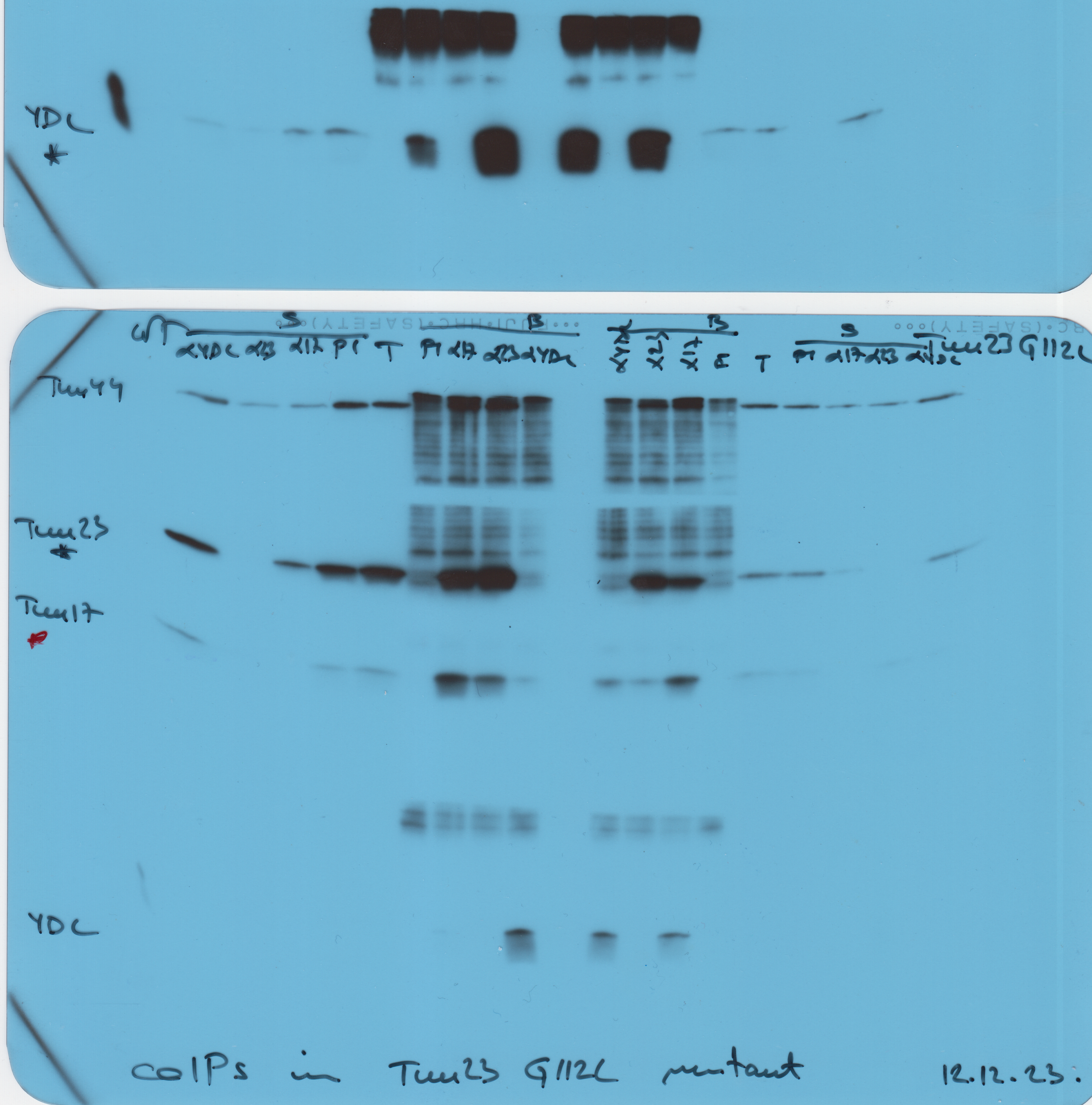

Supplement: Supplementary file 3 — Source data Fig. 2 [file 44319_2024_349_MOESM3_ESM.zip › Fig 2/2C/Co-IP G112L.tiff]

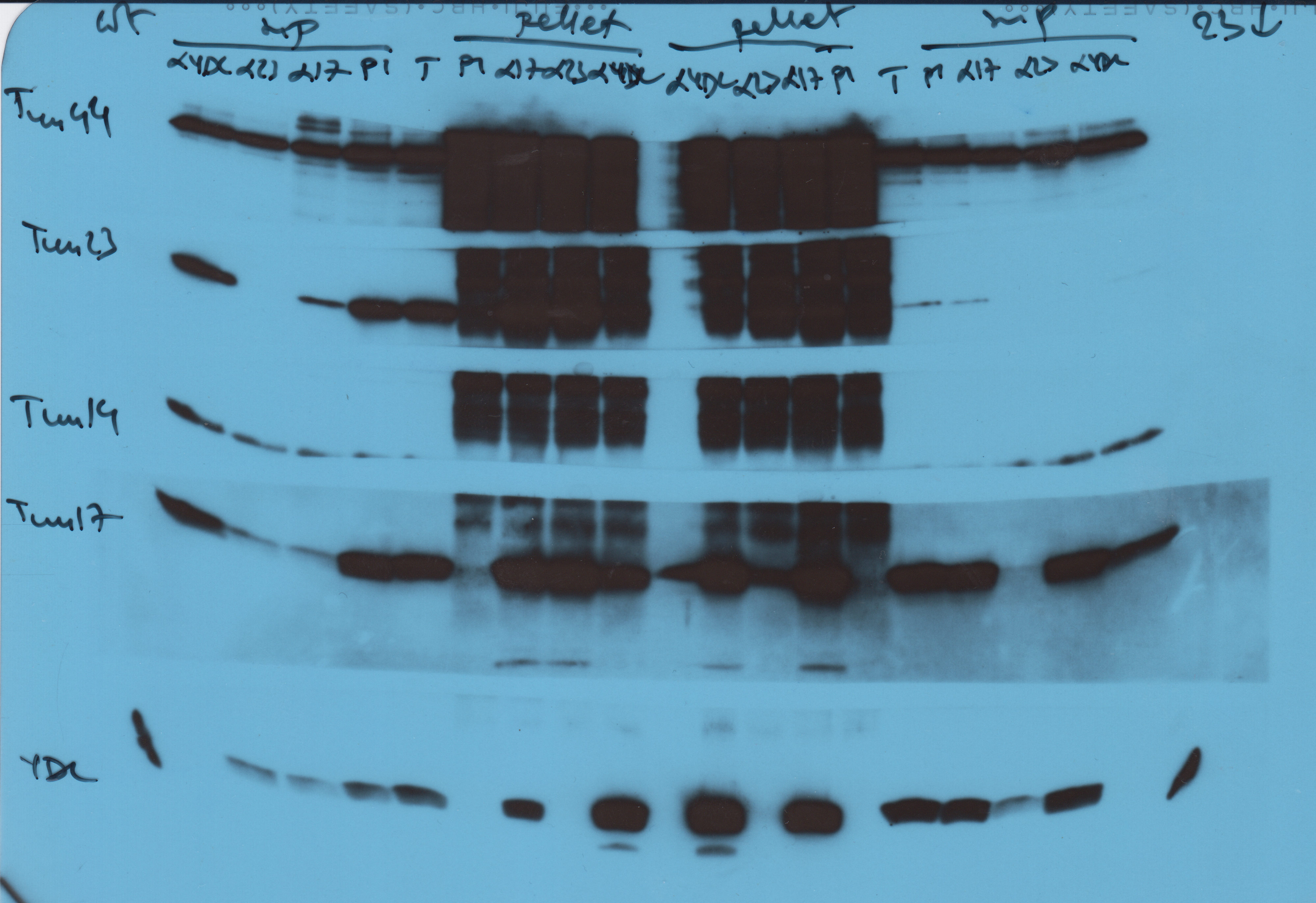

Supplement: Supplementary file 3 — Source data Fig. 2 [file 44319_2024_349_MOESM3_ESM.zip › Fig 2/2D/Co-IP 23 down bound Dbi1.tiff]

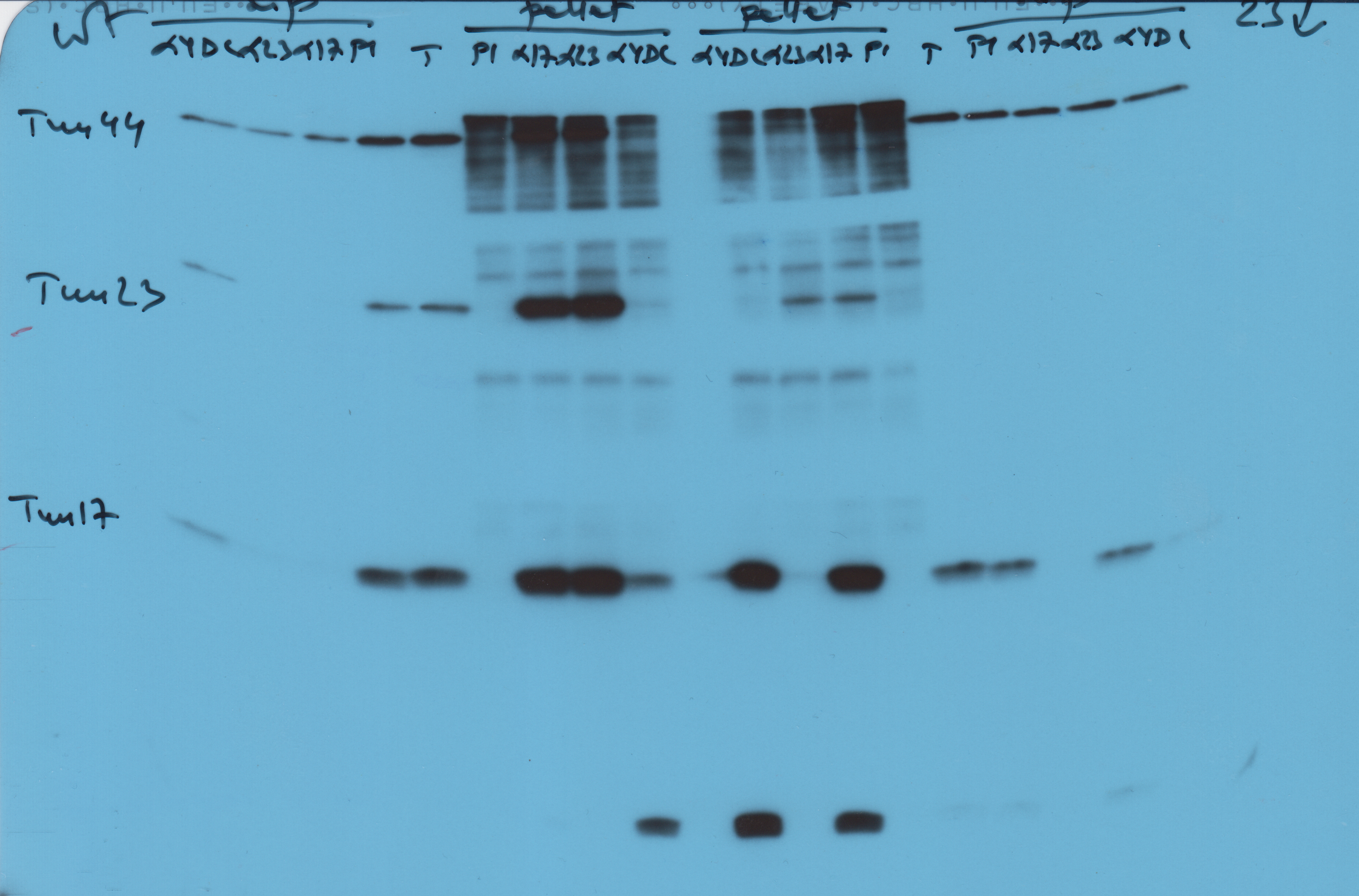

Supplement: Supplementary file 3 — Source data Fig. 2 [file 44319_2024_349_MOESM3_ESM.zip › Fig 2/2D/Co-IP 23 down bound Tim23 tim17.tiff]

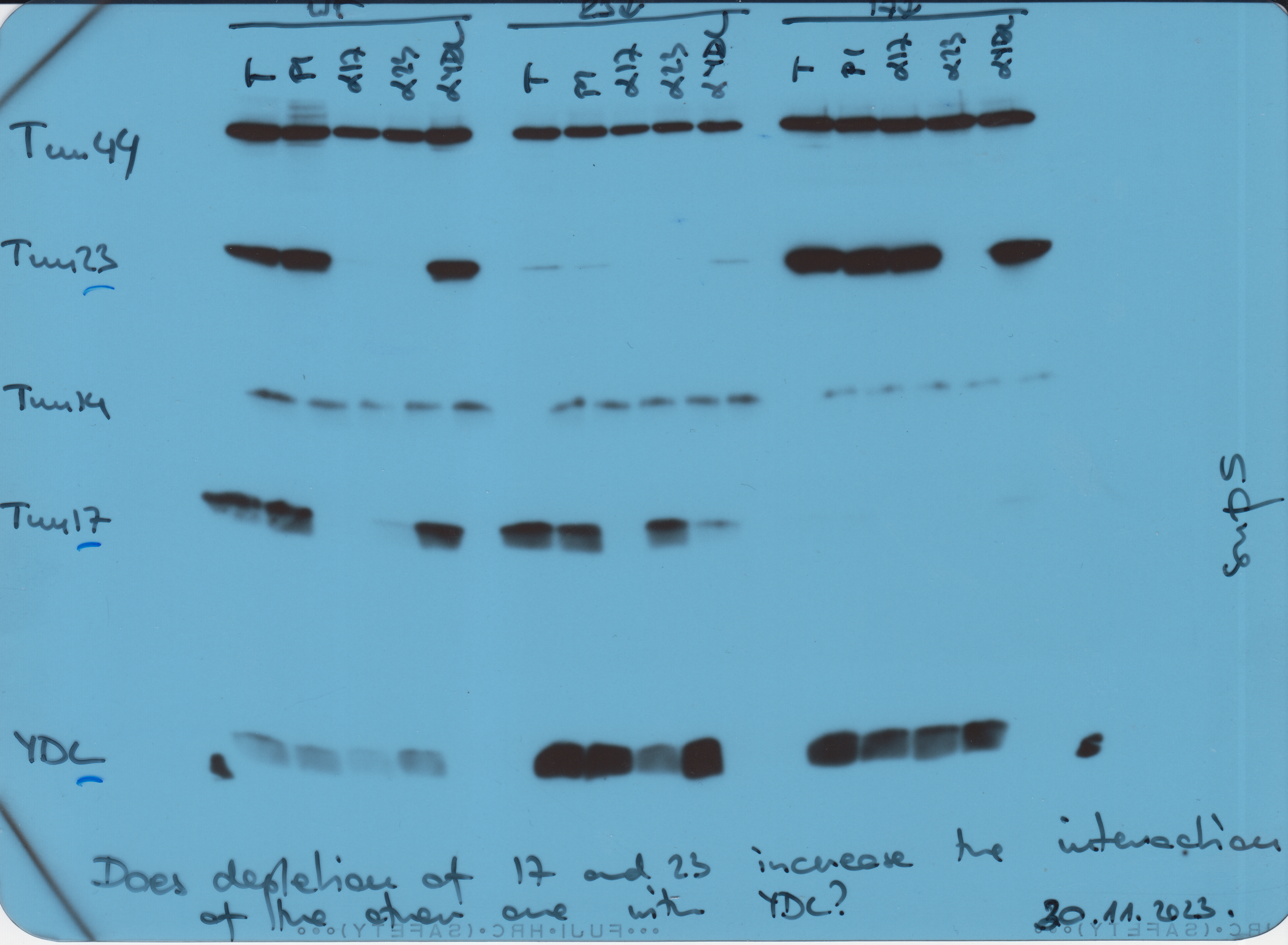

Supplement: Supplementary file 3 — Source data Fig. 2 [file 44319_2024_349_MOESM3_ESM.zip › Fig 2/2E/Co-IP 23 down nb.tiff]

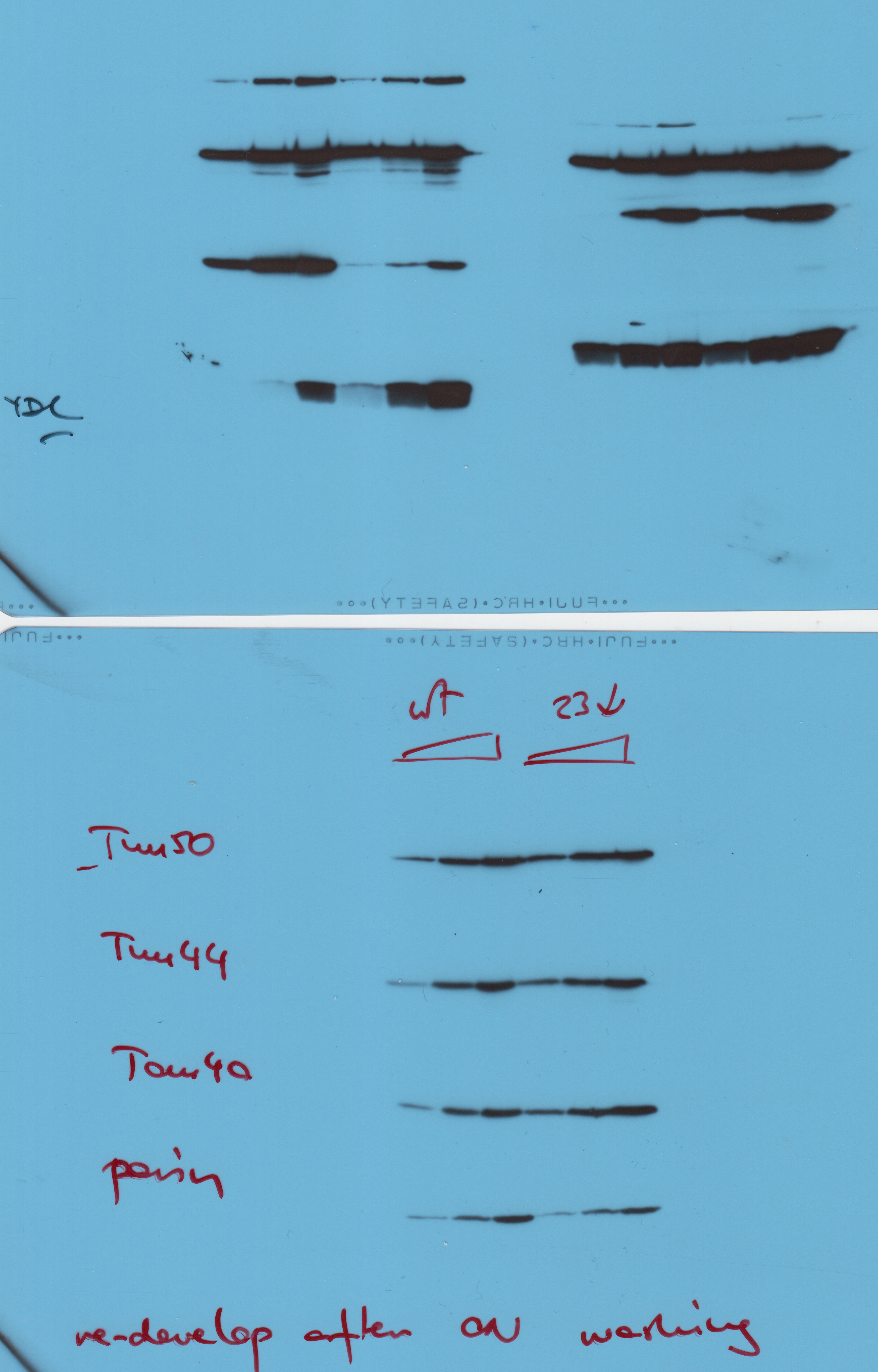

Supplement: Supplementary file 3 — Source data Fig. 2 [file 44319_2024_349_MOESM3_ESM.zip › Fig 2/2F/Mitoprofile wt 23down Dbi1 Tim50.tiff]

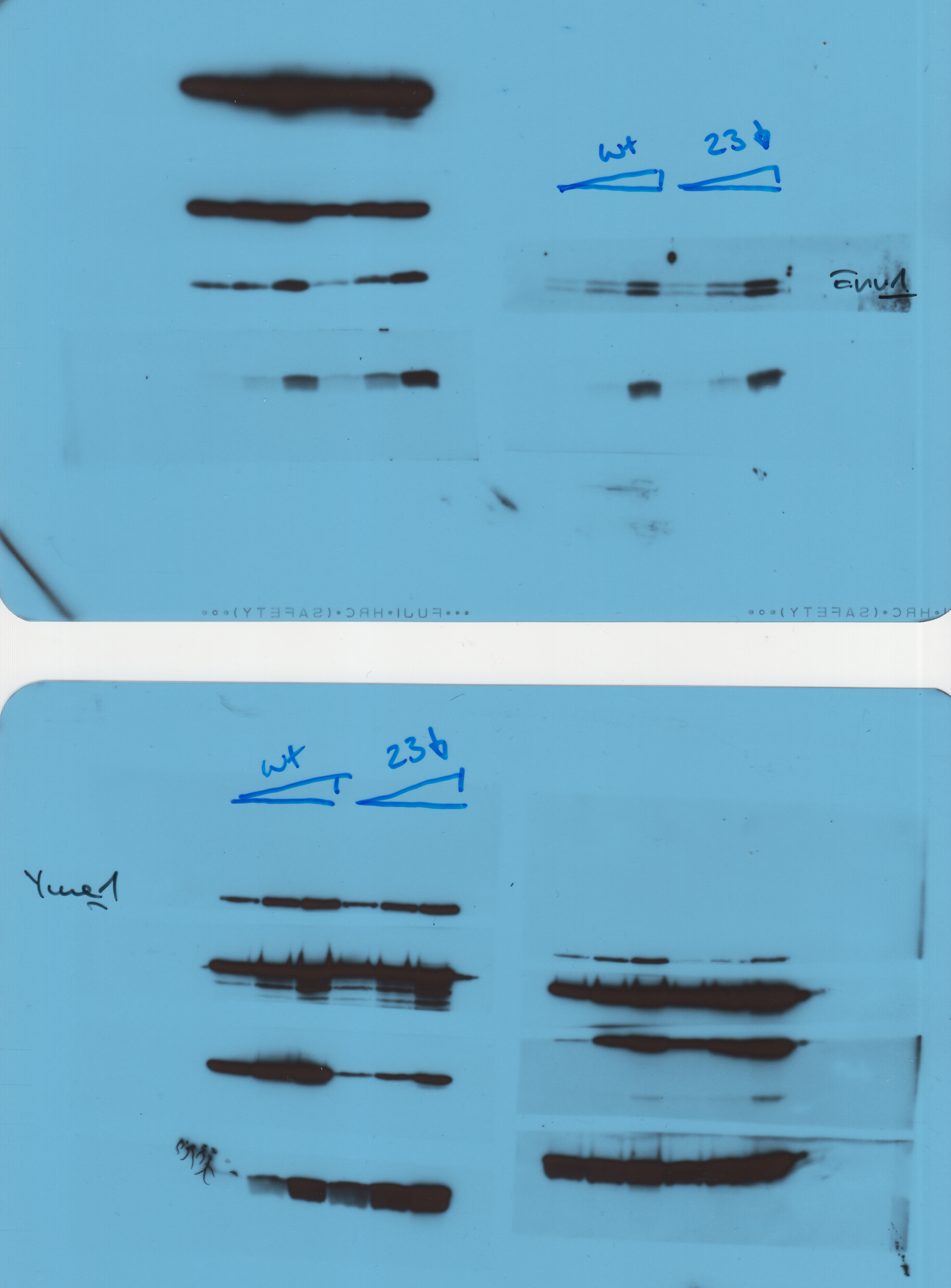

Supplement: Supplementary file 3 — Source data Fig. 2 [file 44319_2024_349_MOESM3_ESM.zip › Fig 2/2F/Mitoprofile wt 23down Erv1 Yme1.tiff]

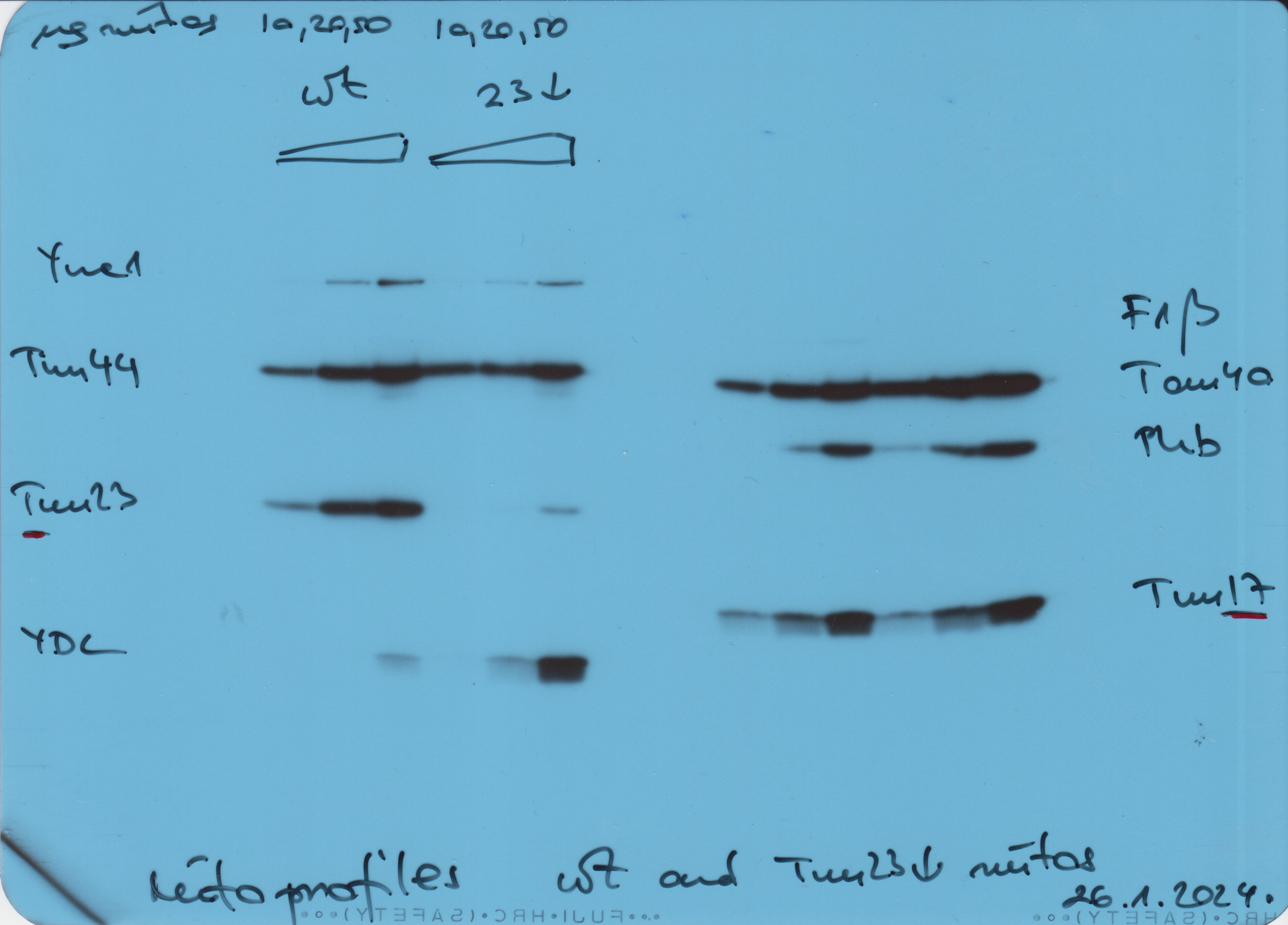

Supplement: Supplementary file 3 — Source data Fig. 2 [file 44319_2024_349_MOESM3_ESM.zip › Fig 2/2F/Mitoprofile wt 23down Tim17 Tim23.tiff]

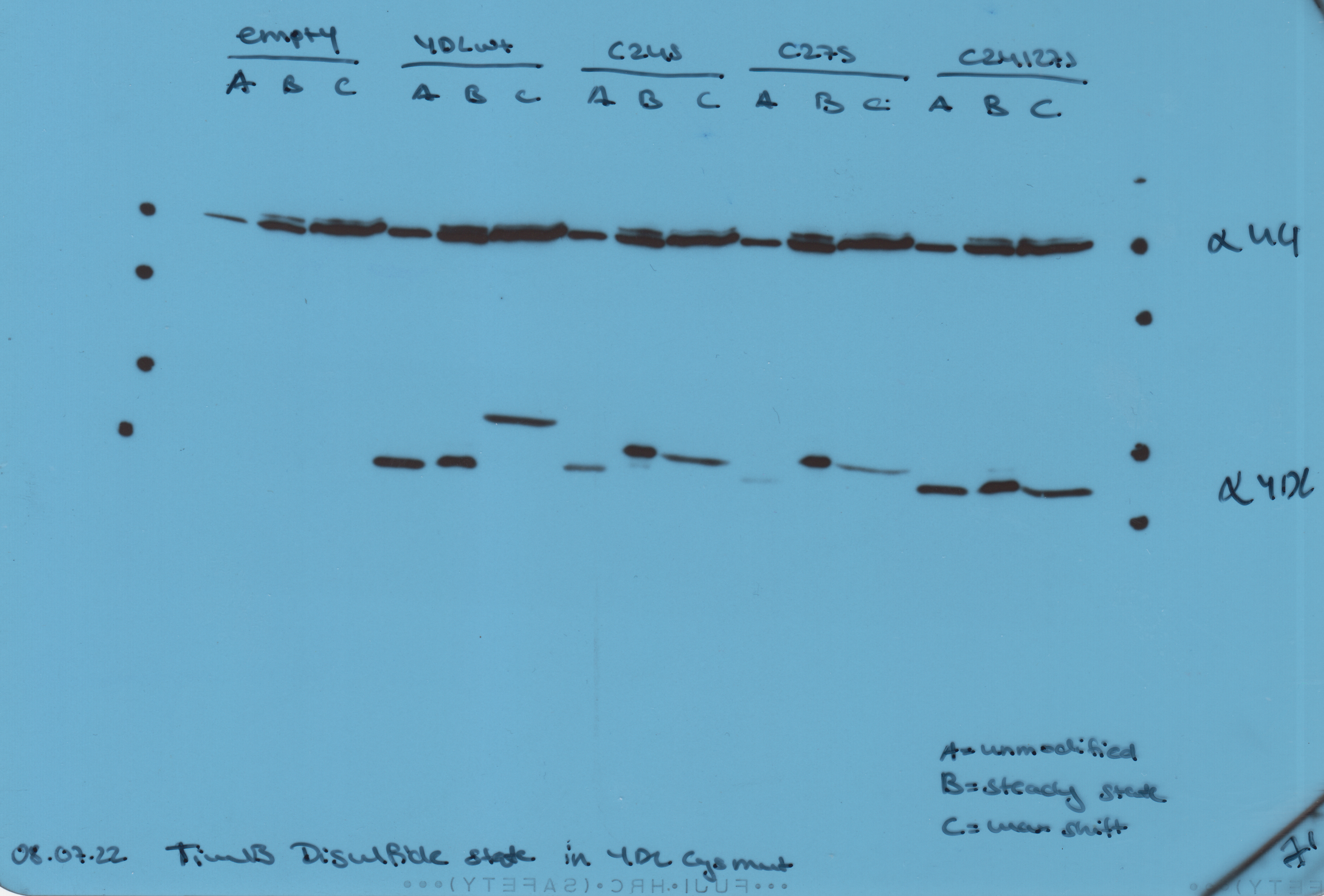

Supplement: Supplementary file 4 — Source data Fig. 3 [file 44319_2024_349_MOESM4_ESM.zip › Fig 3/3B/shift Dbi.tiff]

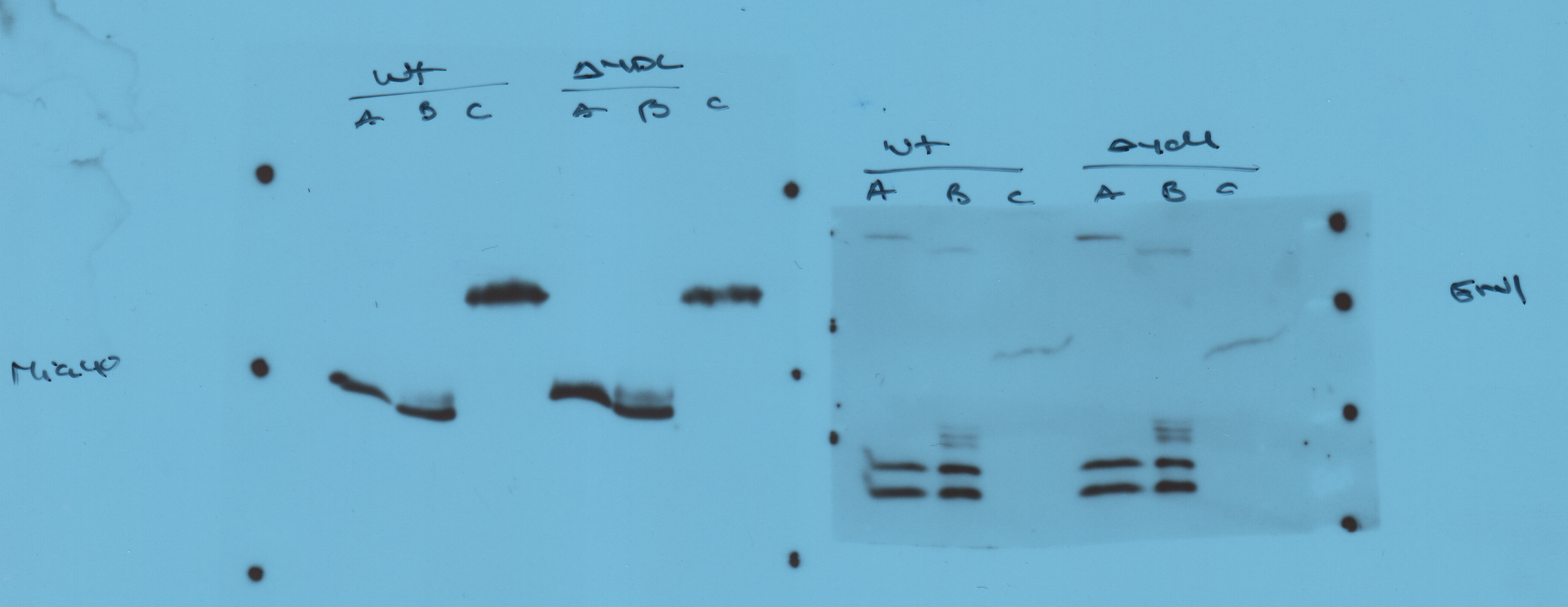

Supplement: Supplementary file 4 — Source data Fig. 3 [file 44319_2024_349_MOESM4_ESM.zip › Fig 3/3C/shift wt delta mia erv.tiff]

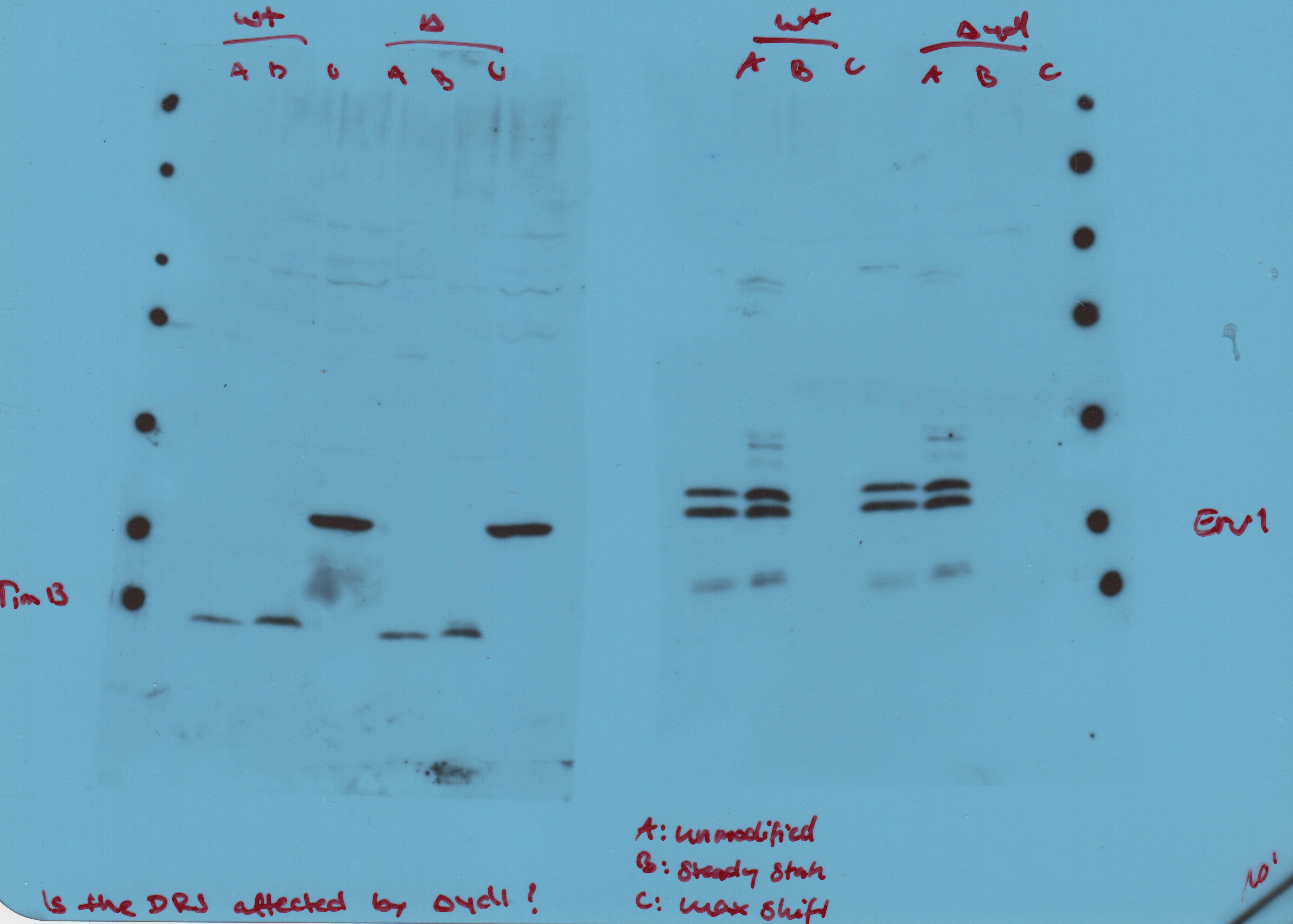

Supplement: Supplementary file 4 — Source data Fig. 3 [file 44319_2024_349_MOESM4_ESM.zip › Fig 3/3C/shift wt delta tim13.tiff]

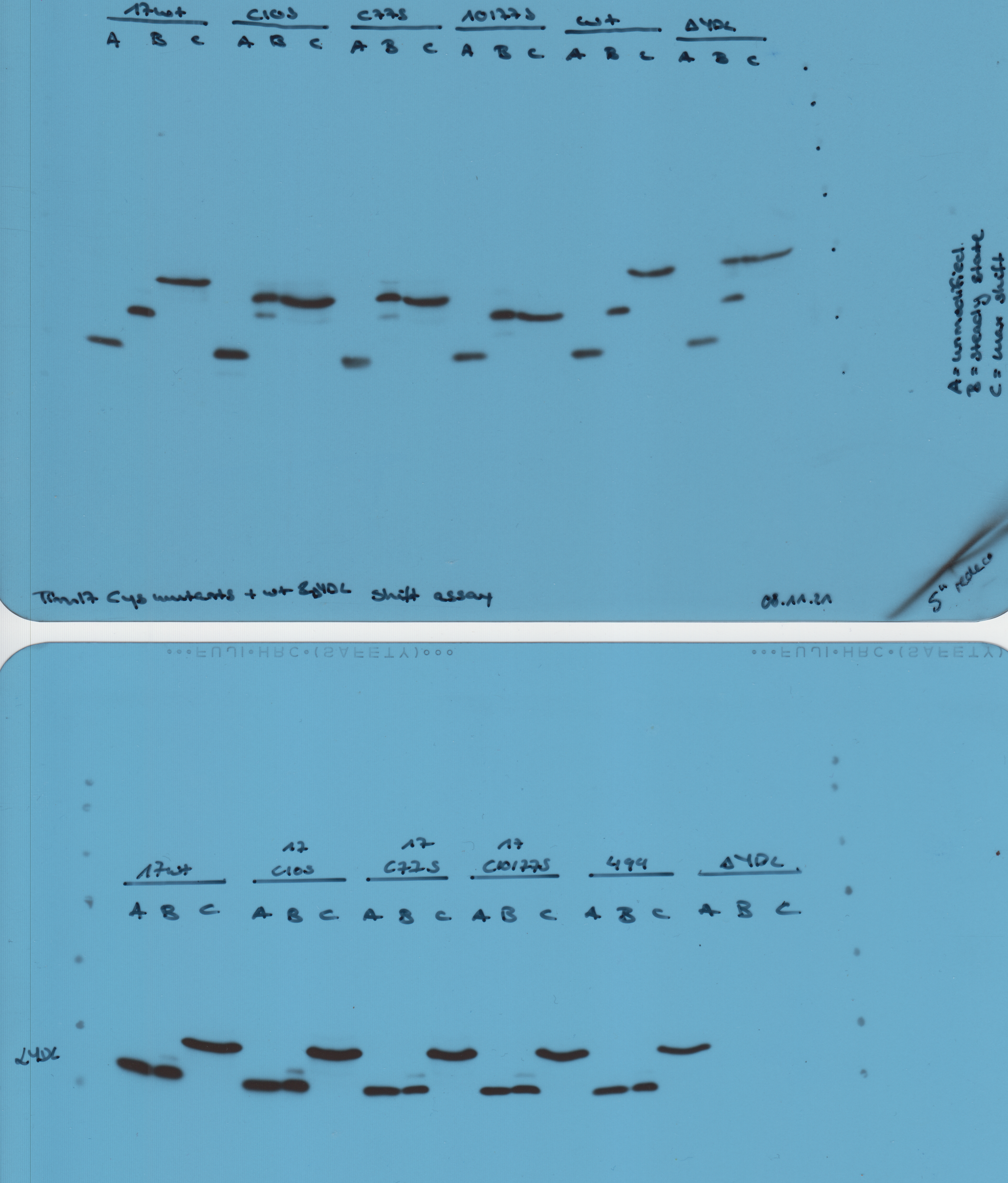

Supplement: Supplementary file 4 — Source data Fig. 3 [file 44319_2024_349_MOESM4_ESM.zip › Fig 3/3C/shift wt delta Tim17 dbi.tiff]

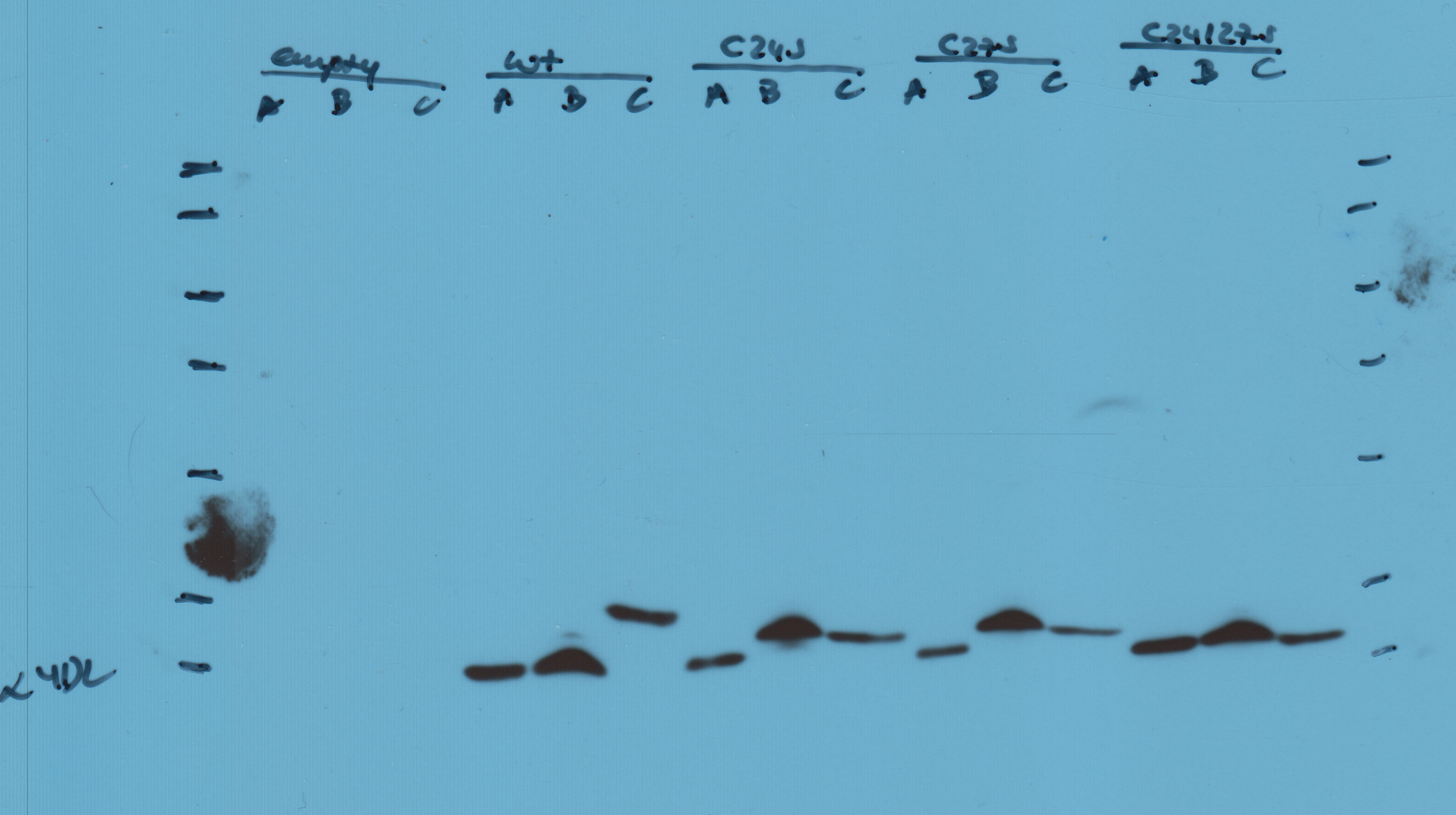

Supplement: Supplementary file 4 — Source data Fig. 3 [file 44319_2024_349_MOESM4_ESM.zip › Fig 3/3D/shift Dbi Cys Dbi1.tiff]

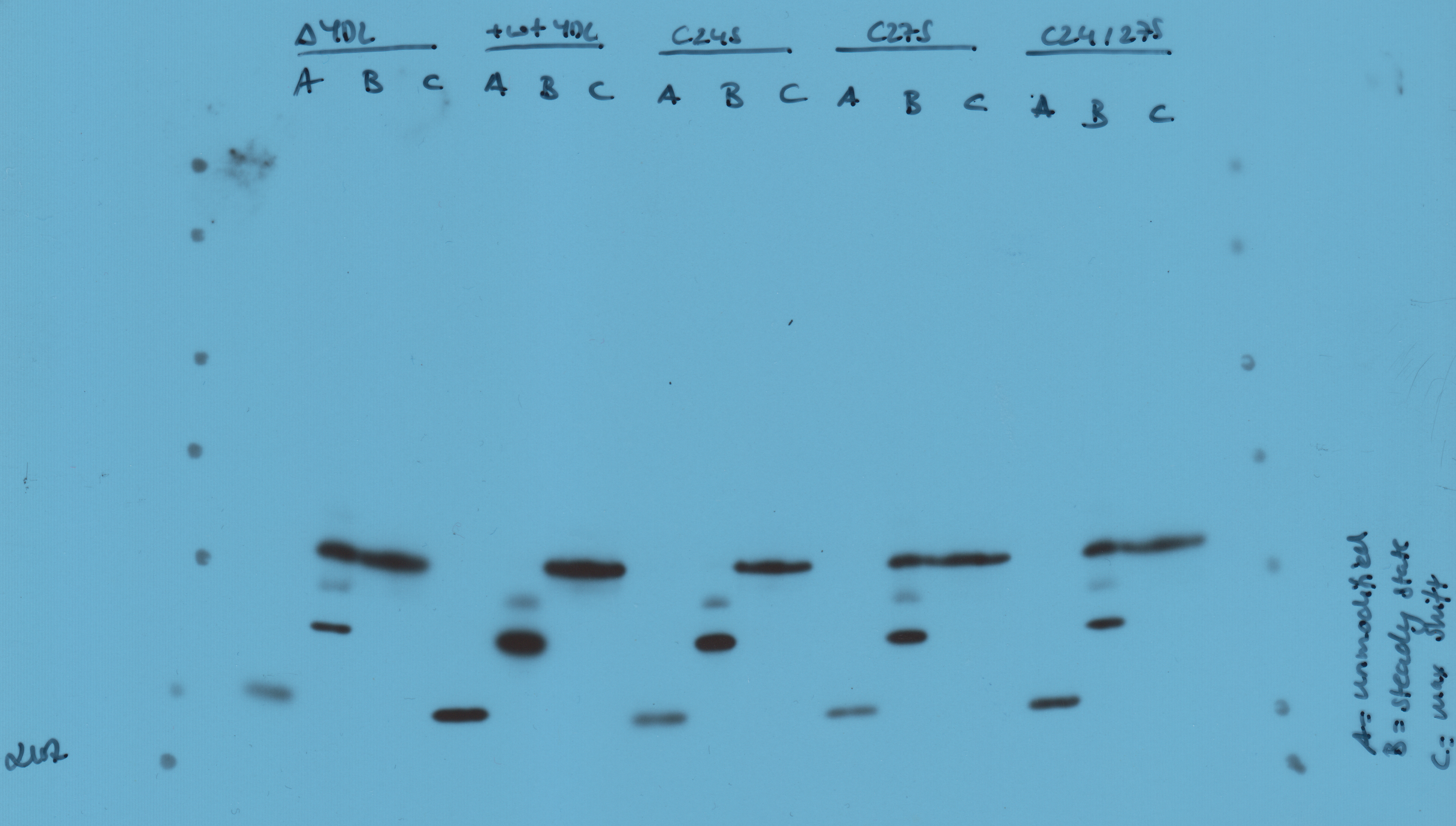

Supplement: Supplementary file 4 — Source data Fig. 3 [file 44319_2024_349_MOESM4_ESM.zip › Fig 3/3D/shift Dbi Cys Tim17 lower blott.tiff]

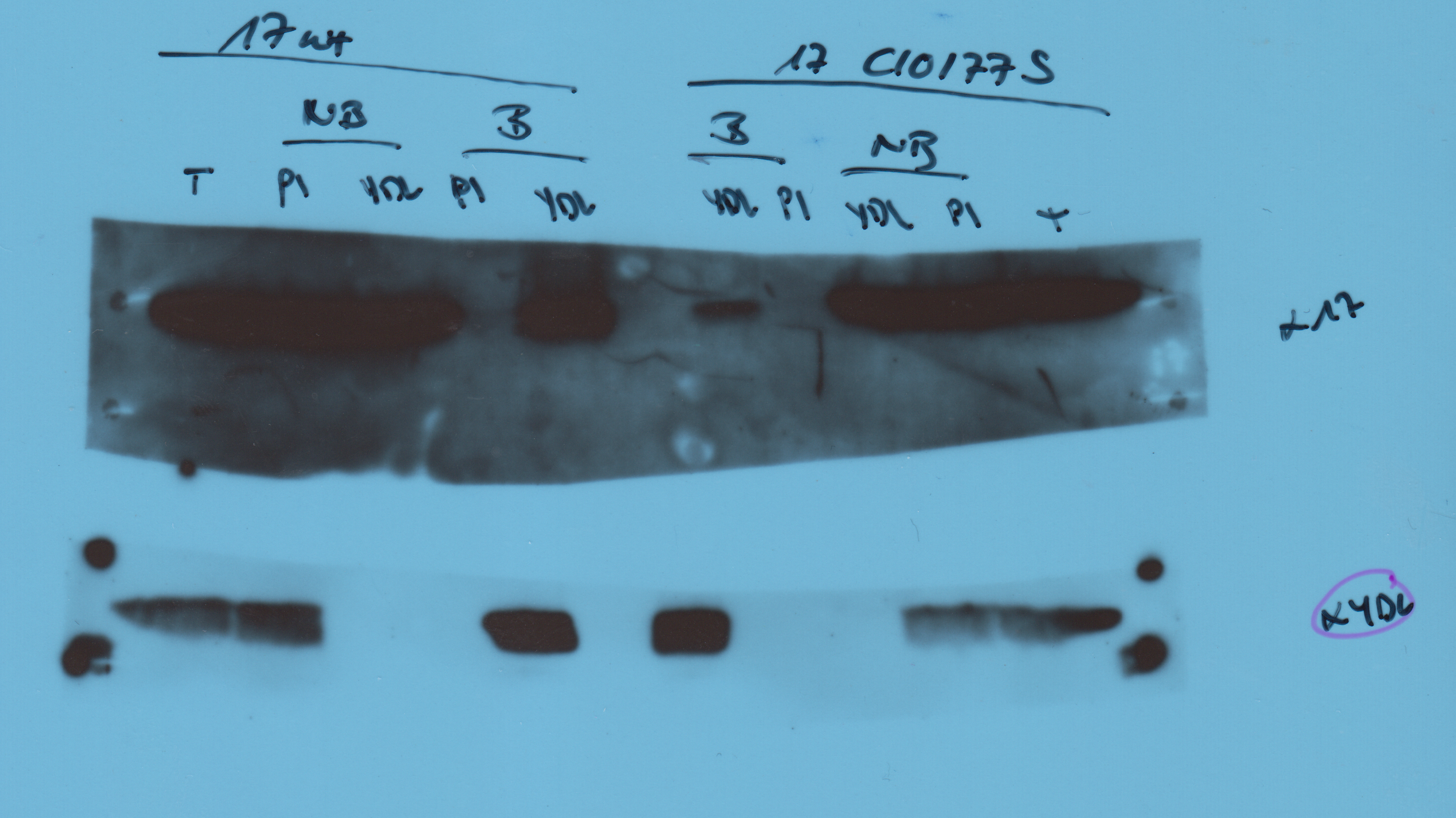

Supplement: Supplementary file 4 — Source data Fig. 3 [file 44319_2024_349_MOESM4_ESM.zip › Fig 3/3E/Co-IP Dbi1.tiff]

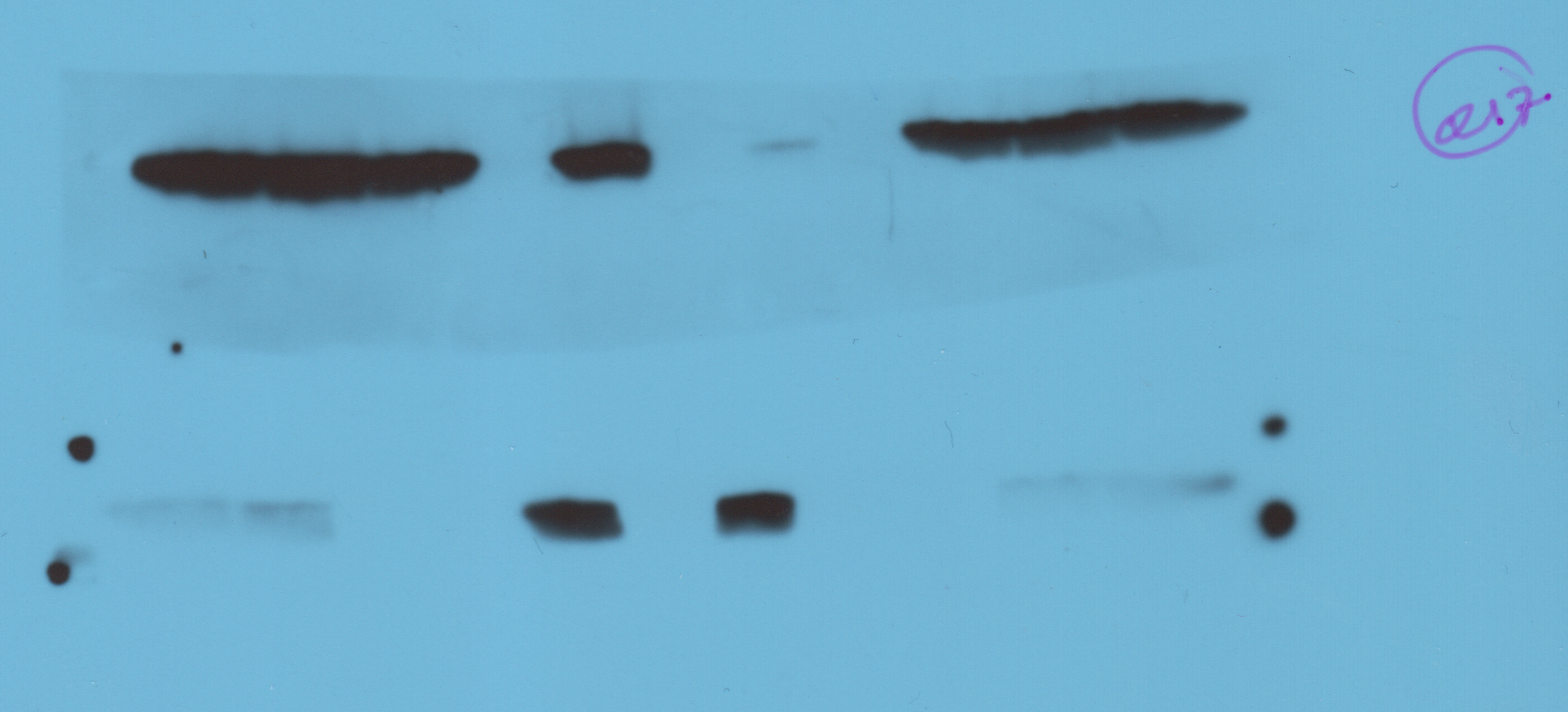

Supplement: Supplementary file 4 — Source data Fig. 3 [file 44319_2024_349_MOESM4_ESM.zip › Fig 3/3E/Co-IP Tim17.tiff]

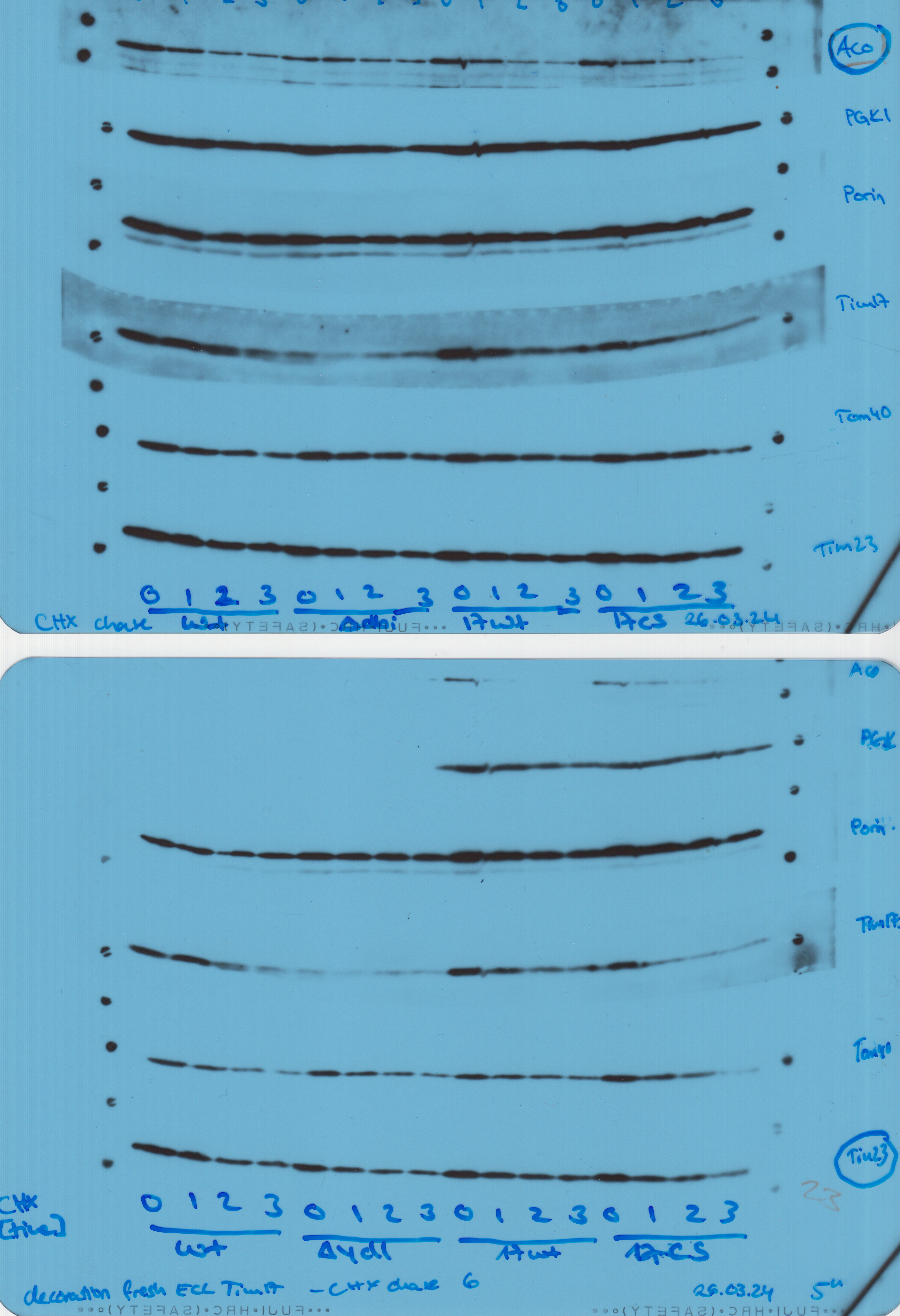

Supplement: Supplementary file 4 — Source data Fig. 3 [file 44319_2024_349_MOESM4_ESM.zip › Fig 3/3F/CHX Aco1 Tim23.tiff]

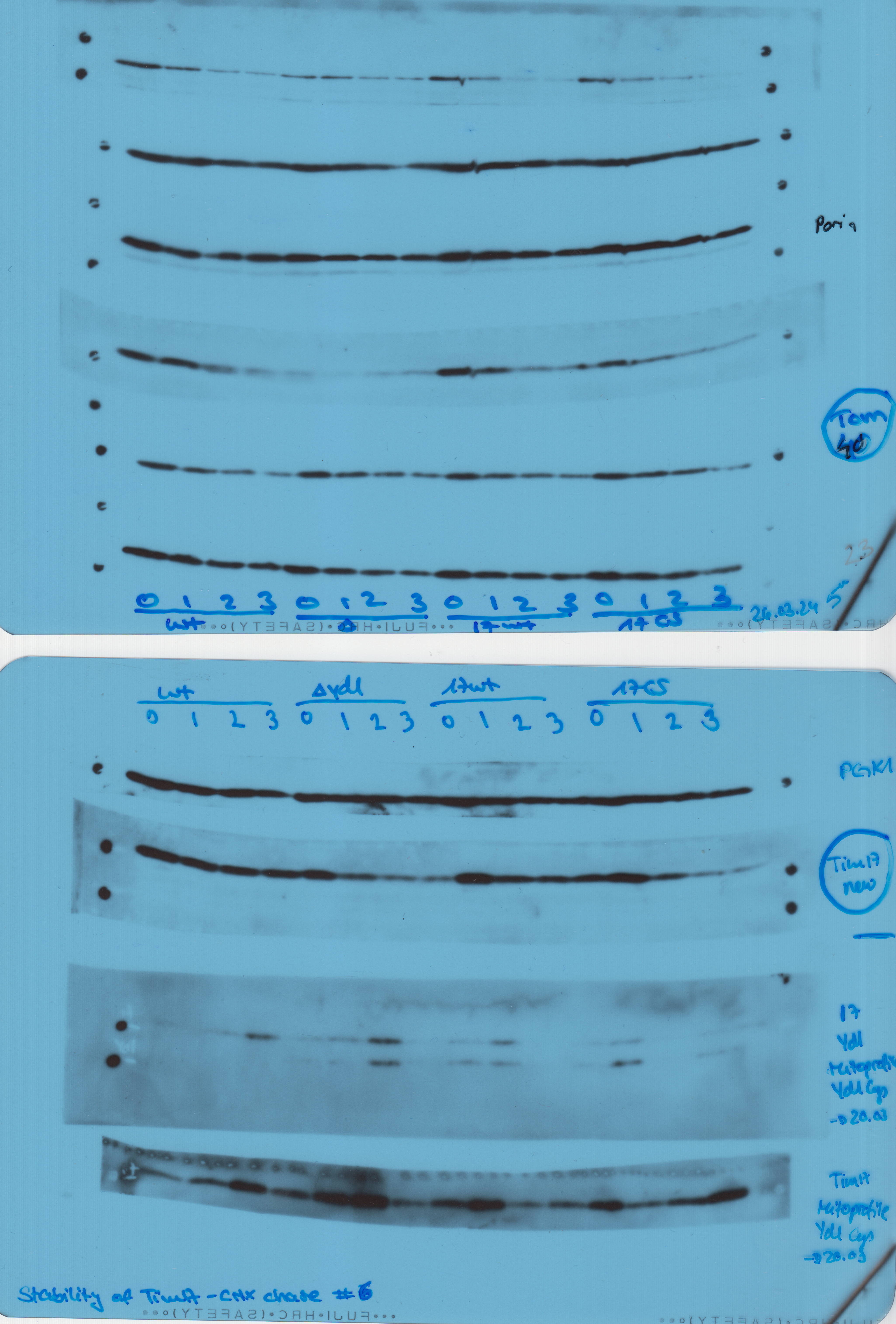

Supplement: Supplementary file 4 — Source data Fig. 3 [file 44319_2024_349_MOESM4_ESM.zip › Fig 3/3F/CHX Tom40 Tim17.tiff]

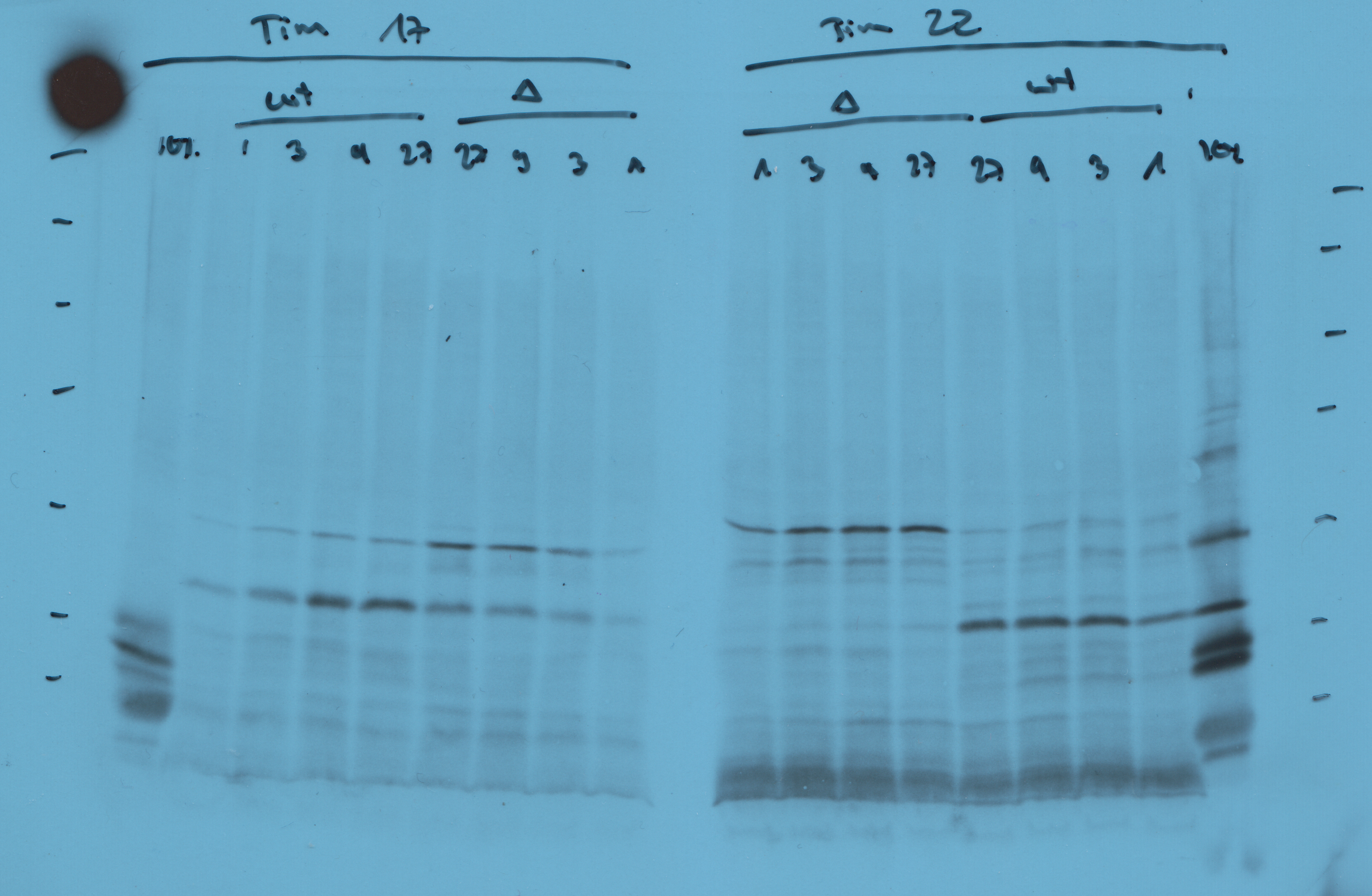

Supplement: Supplementary file 4 — Source data Fig. 3 [file 44319_2024_349_MOESM4_ESM.zip › Fig 3/3G/Import and shift Tim17.tiff]

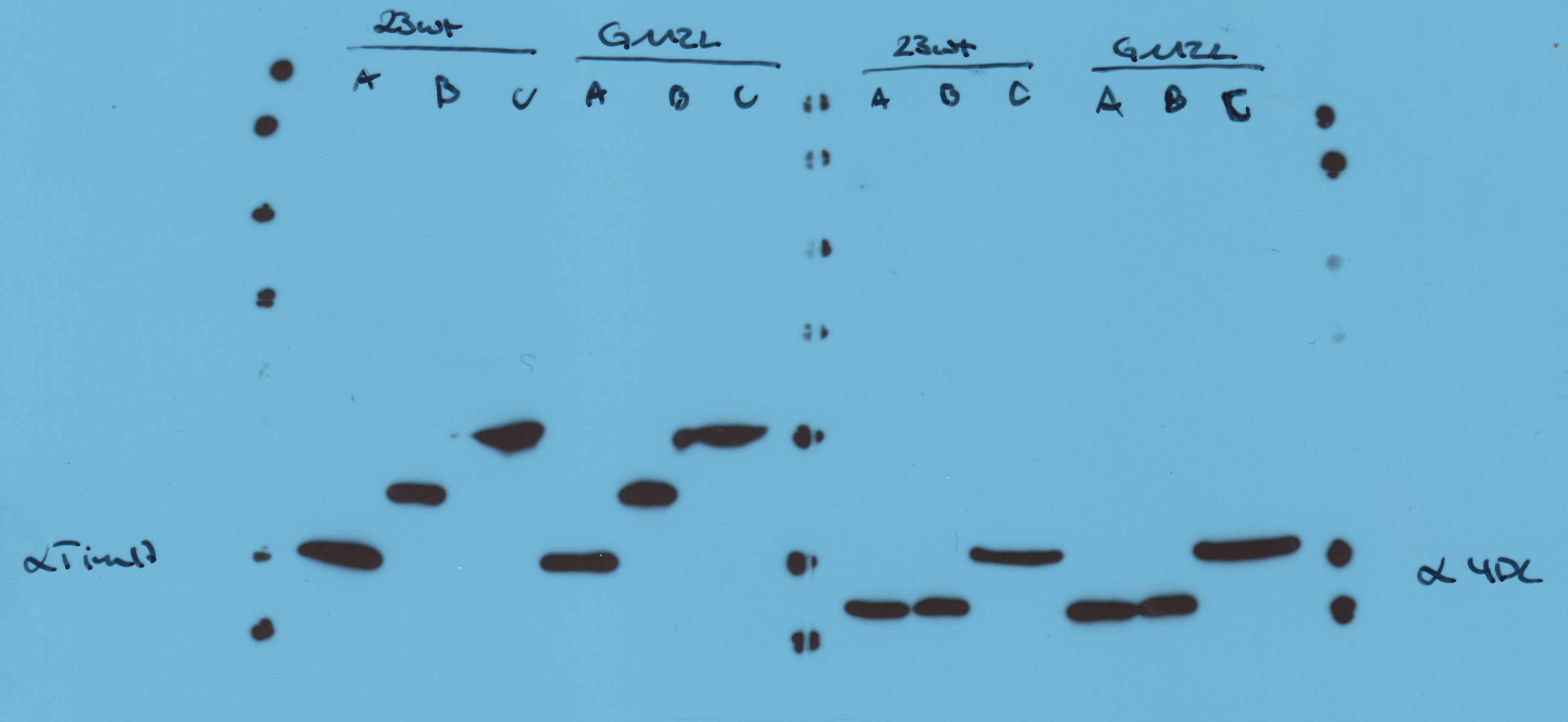

Supplement: Supplementary file 4 — Source data Fig. 3 [file 44319_2024_349_MOESM4_ESM.zip › Fig 3/3H/shift G112L Dbi1.tiff]

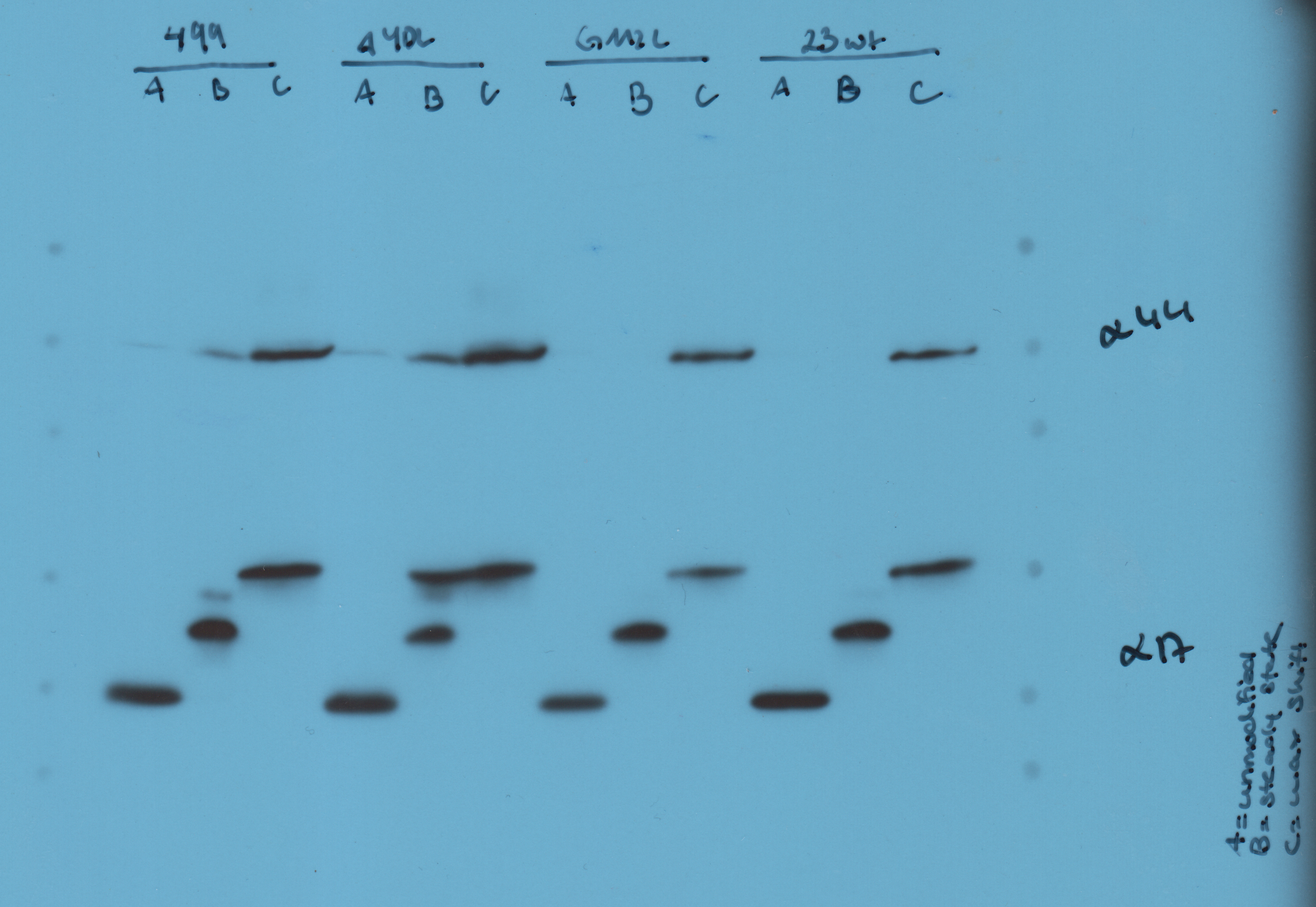

Supplement: Supplementary file 4 — Source data Fig. 3 [file 44319_2024_349_MOESM4_ESM.zip › Fig 3/3H/shift G112L Tim17.tiff]

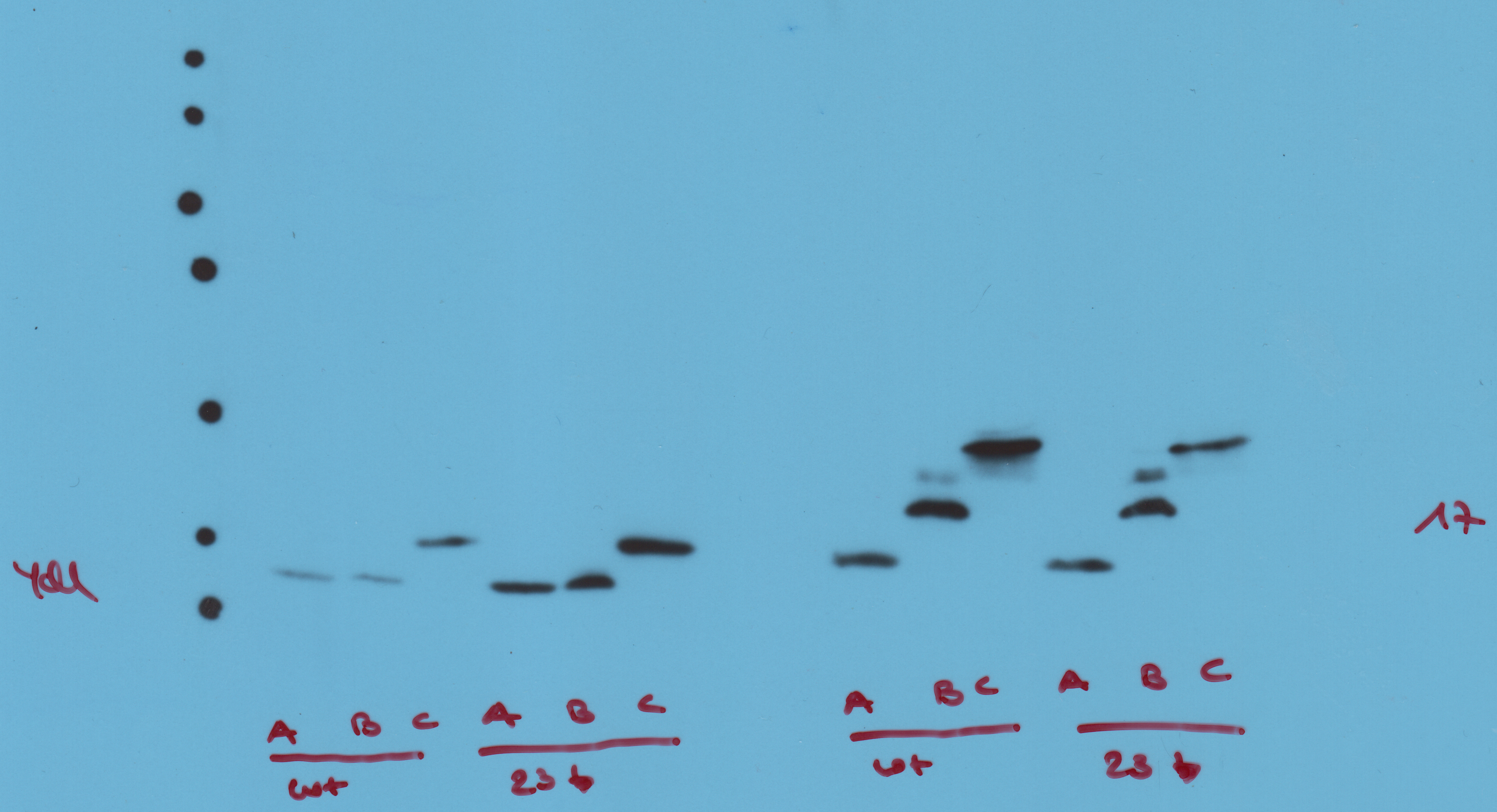

Supplement: Supplementary file 4 — Source data Fig. 3 [file 44319_2024_349_MOESM4_ESM.zip › Fig 3/3I/shift 23 down Dbi1 and Tim17.tiff]

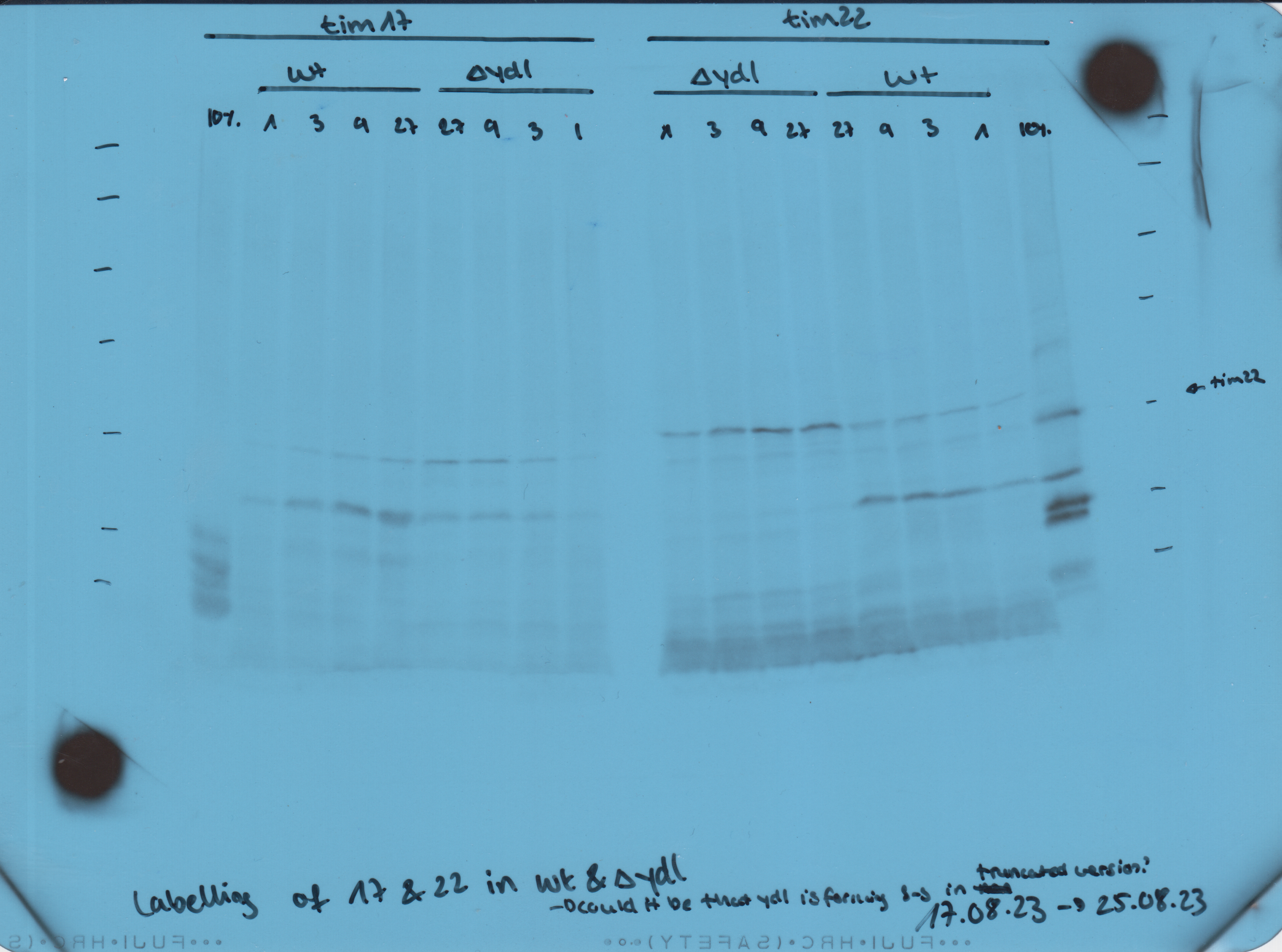

Supplement: Supplementary file 5 — Source data Fig. 4 [file 44319_2024_349_MOESM5_ESM.zip › Fig 4/4B/Import and shift Tim22.tiff]

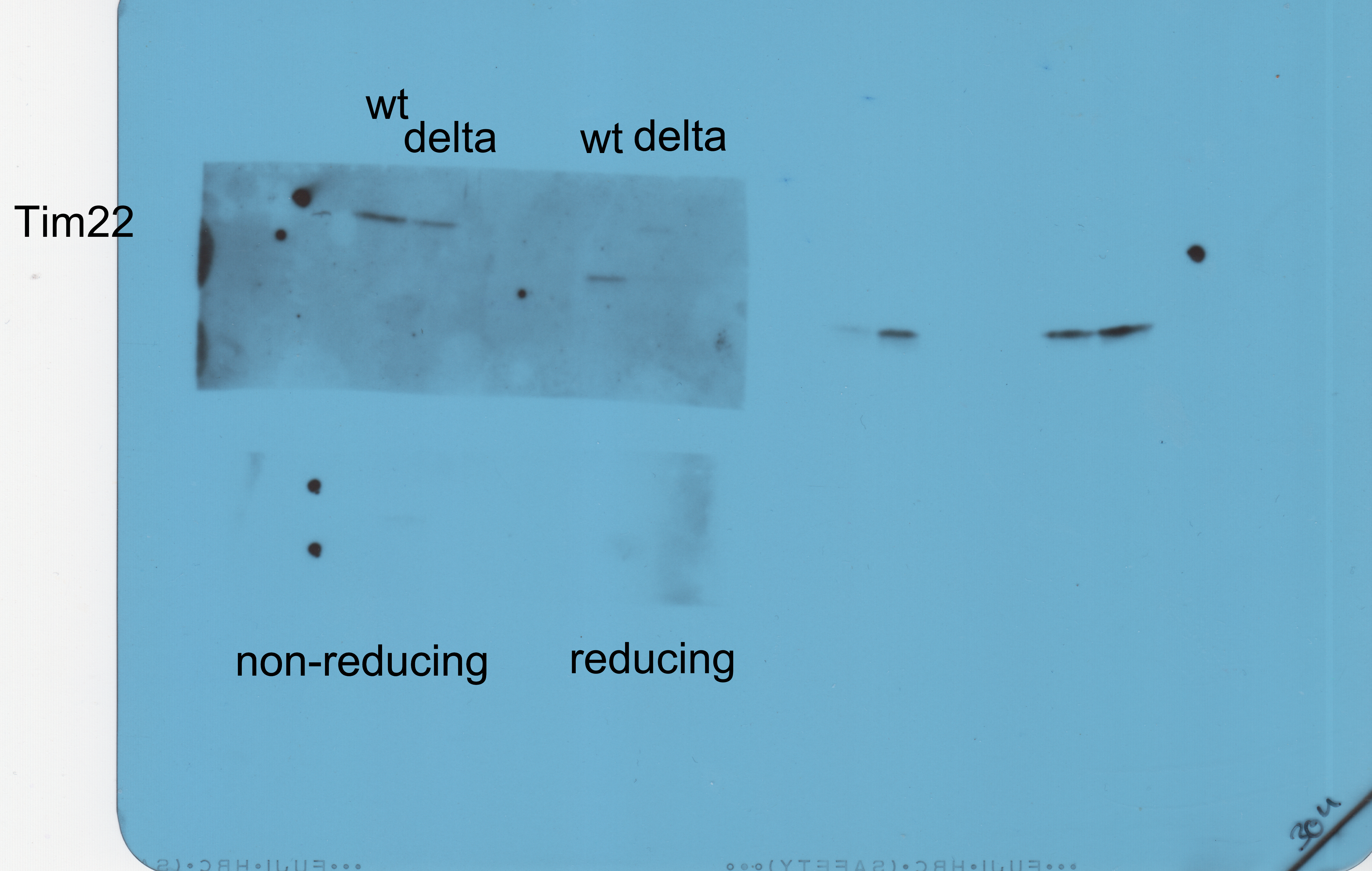

Supplement: Supplementary file 5 — Source data Fig. 4 [file 44319_2024_349_MOESM5_ESM.zip › Fig 4/4C/Tim22 oxidation state wt and delta dbi1_with labels.tif]

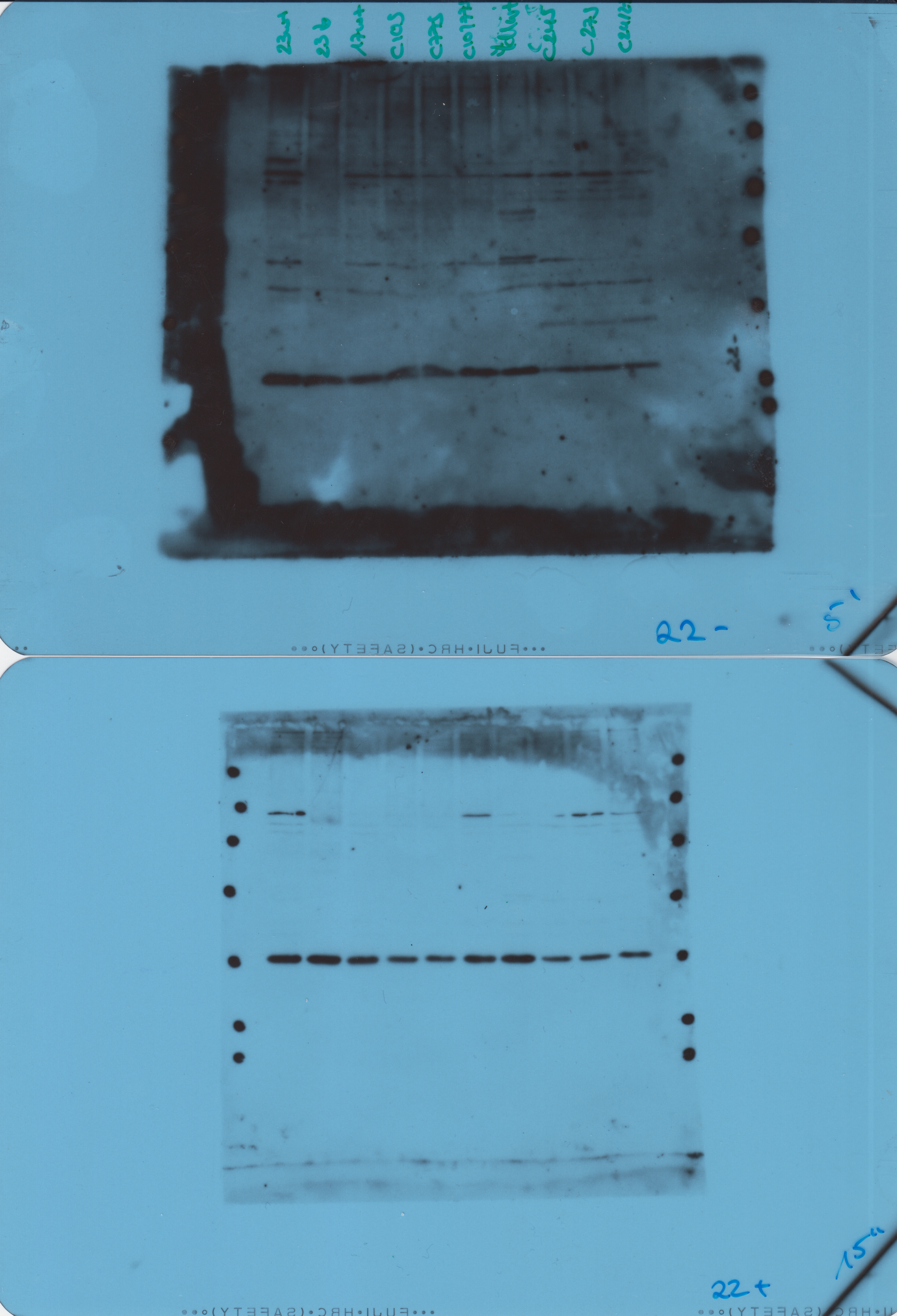

Supplement: Supplementary file 5 — Source data Fig. 4 [file 44319_2024_349_MOESM5_ESM.zip › Fig 4/4D/Tim22 oxidation state Dbi1 Cys mut.tiff]

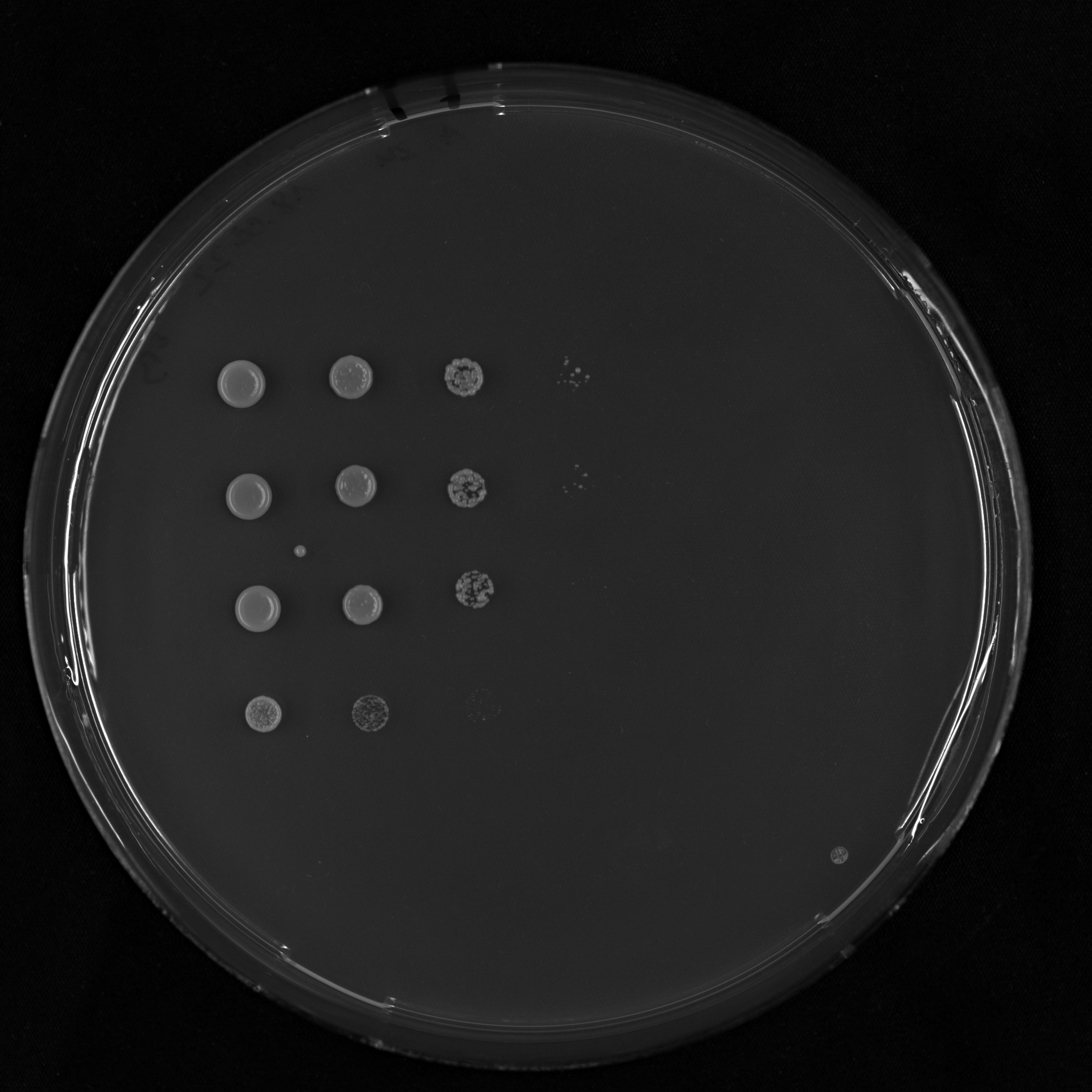

Supplement: Supplementary file 5 — Source data Fig. 4 [file 44319_2024_349_MOESM5_ESM.zip › Fig 4/4E/Drop Dilution Glucose Pam17 Dbi1.Tif]

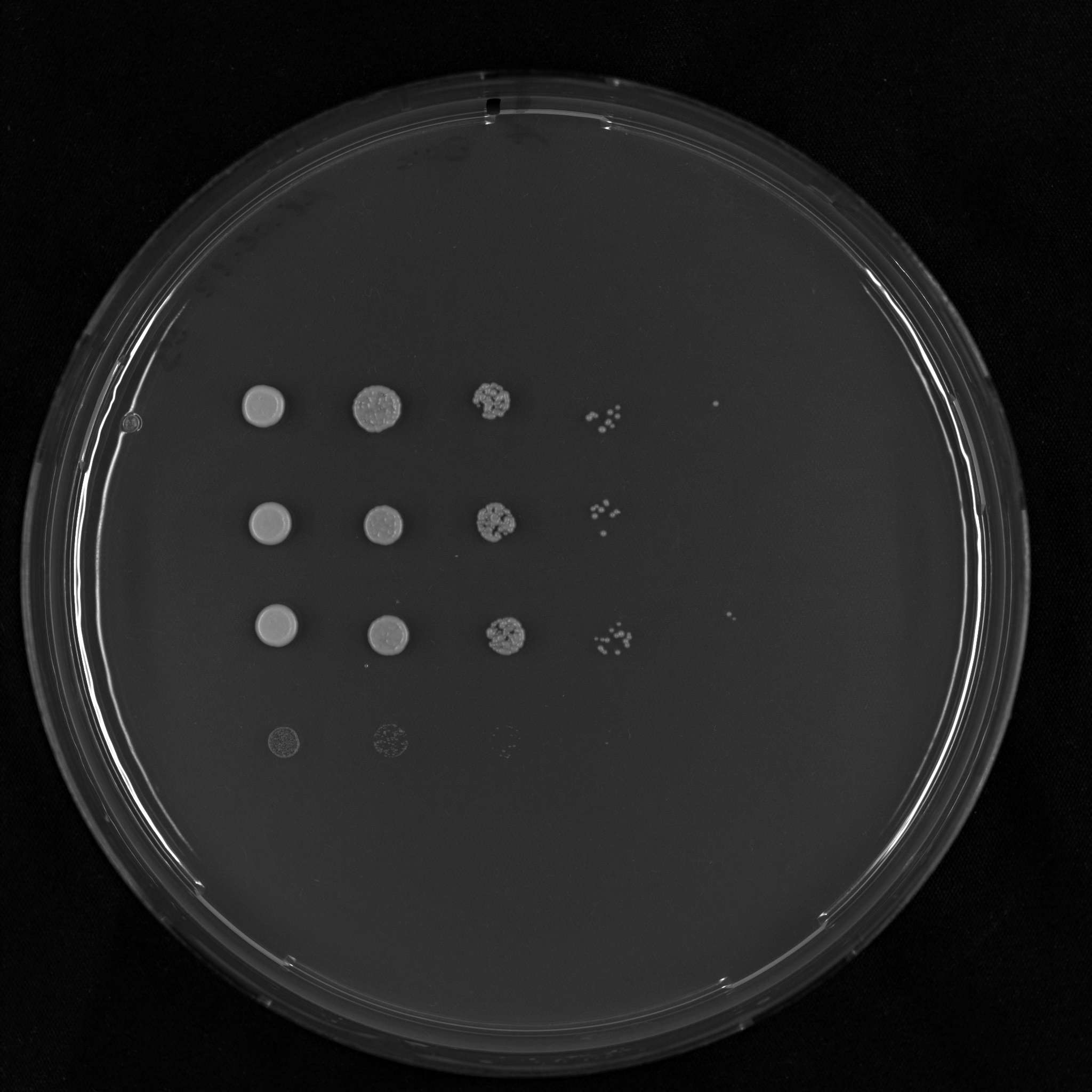

Supplement: Supplementary file 5 — Source data Fig. 4 [file 44319_2024_349_MOESM5_ESM.zip › Fig 4/4E/Drop Dilution Lactate Pam17 dbi1.Tif]

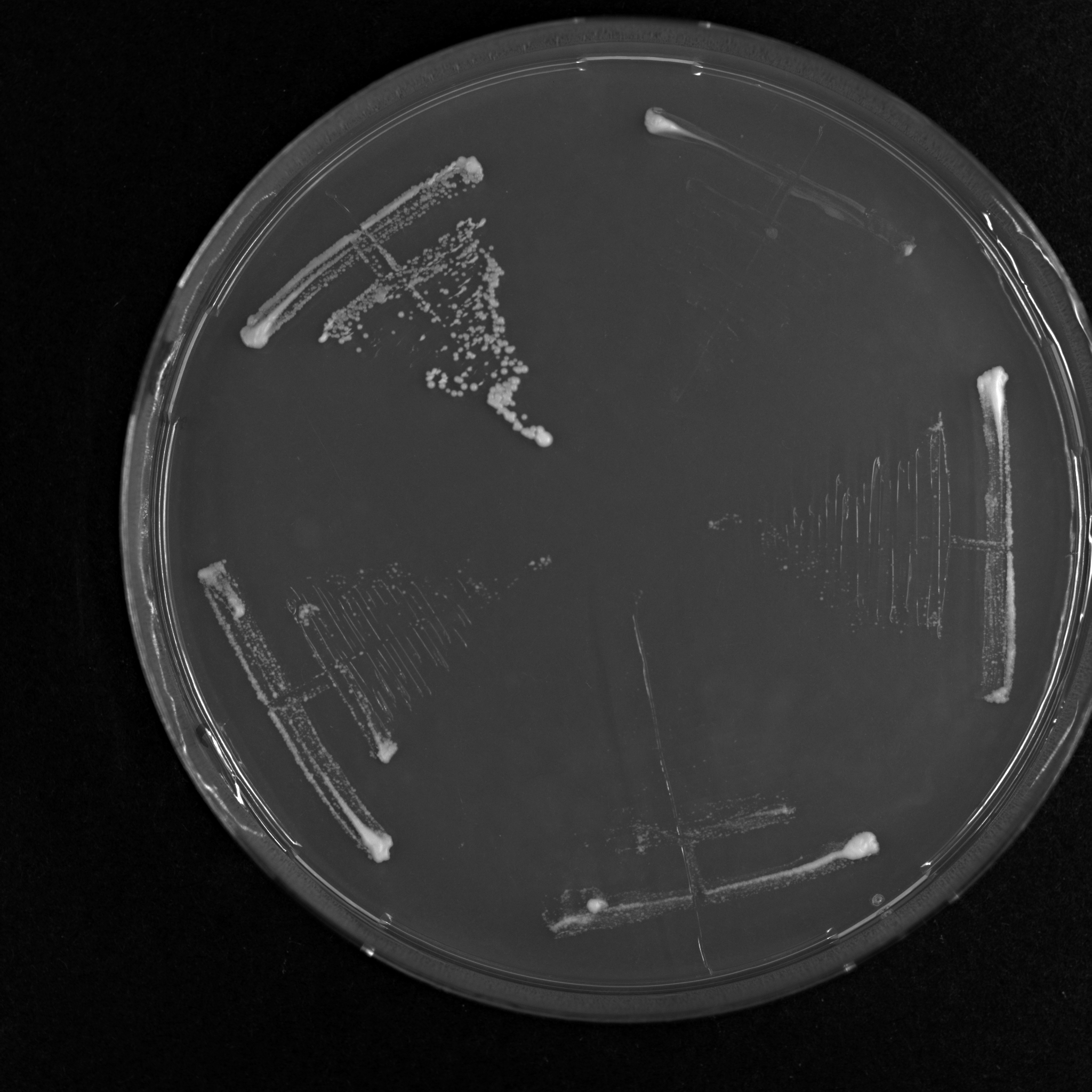

Supplement: Supplementary file 5 — Source data Fig. 4 [file 44319_2024_349_MOESM5_ESM.zip › Fig 4/4F/5FoA Pam17Tim17.Tif]

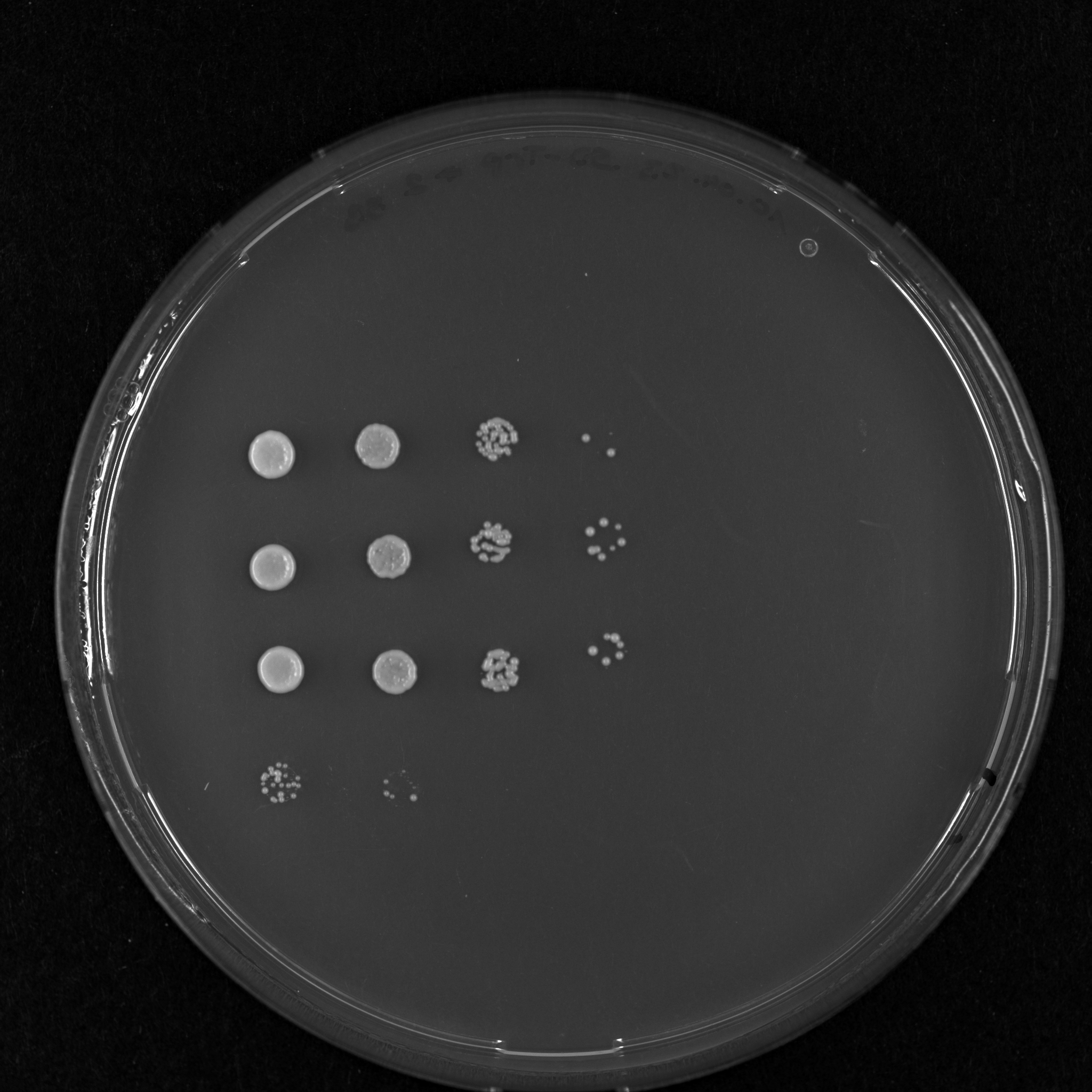

Supplement: Supplementary file 5 — Source data Fig. 4 [file 44319_2024_349_MOESM5_ESM.zip › Fig 4/4G/Drop Dilution Glucose dbi1 tim18 .Tif]

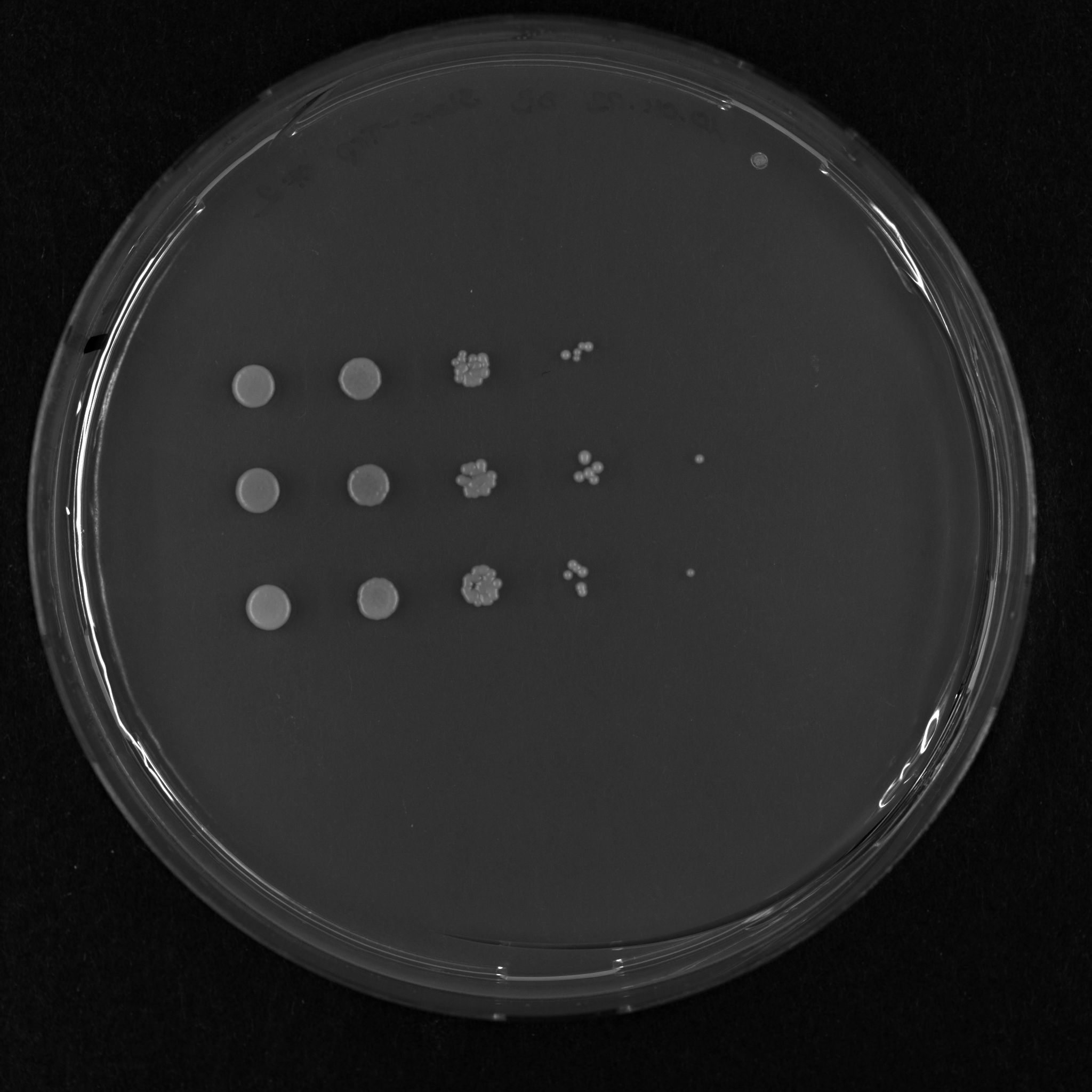

Supplement: Supplementary file 5 — Source data Fig. 4 [file 44319_2024_349_MOESM5_ESM.zip › Fig 4/4G/drop dilution lactate dbi1 tim18.Tif]

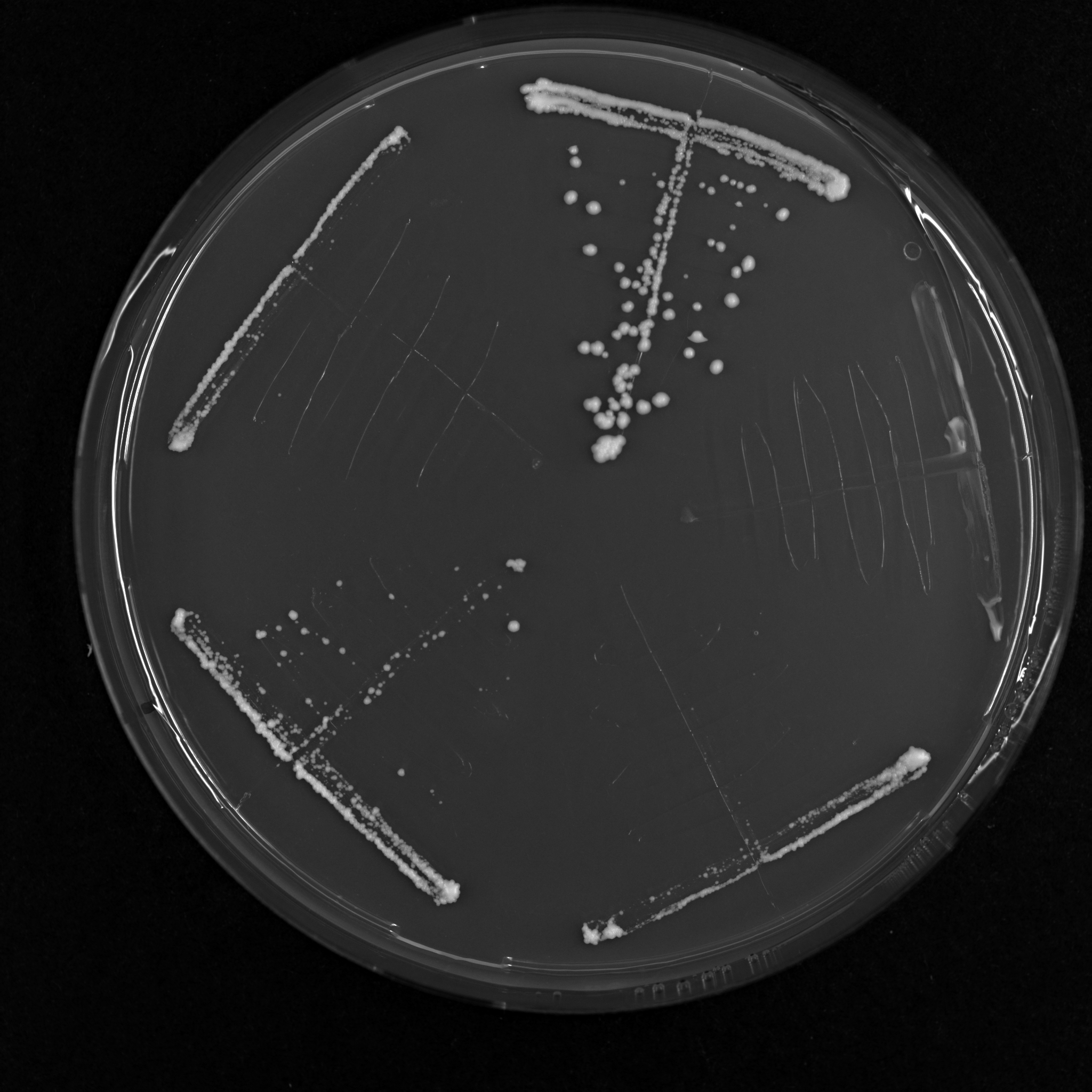

Supplement: Supplementary file 5 — Source data Fig. 4 [file 44319_2024_349_MOESM5_ESM.zip › Fig 4/4H/5FoA Tim18Tim22.Tif]

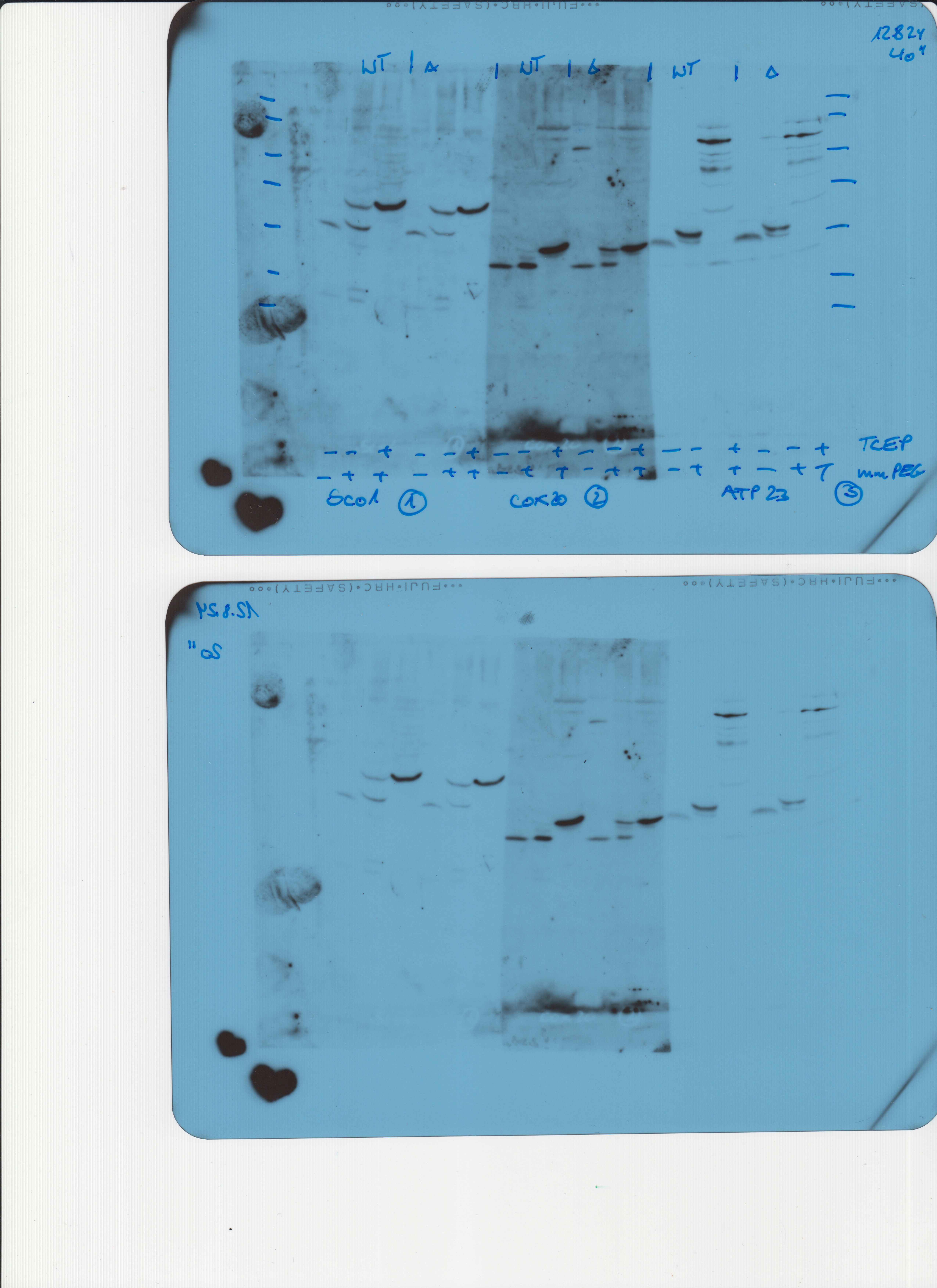

Supplement: Supplementary file 6 — Source data Fig. 5 [file 44319_2024_349_MOESM6_ESM.zip › Fig 5/A/shift wt delta Cox20.tiff]

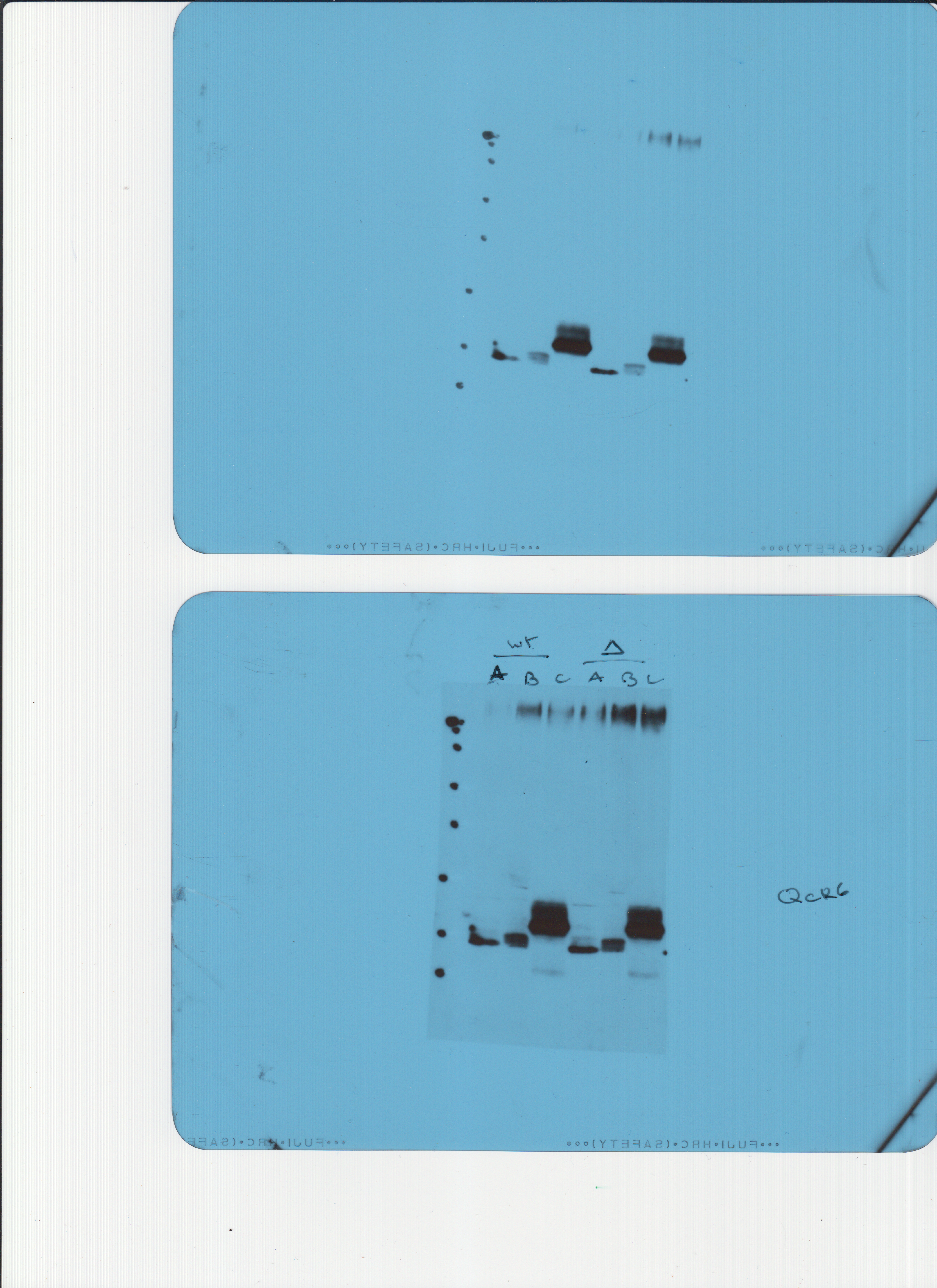

Supplement: Supplementary file 6 — Source data Fig. 5 [file 44319_2024_349_MOESM6_ESM.zip › Fig 5/A/shift wt delta Qcr6.tiff]

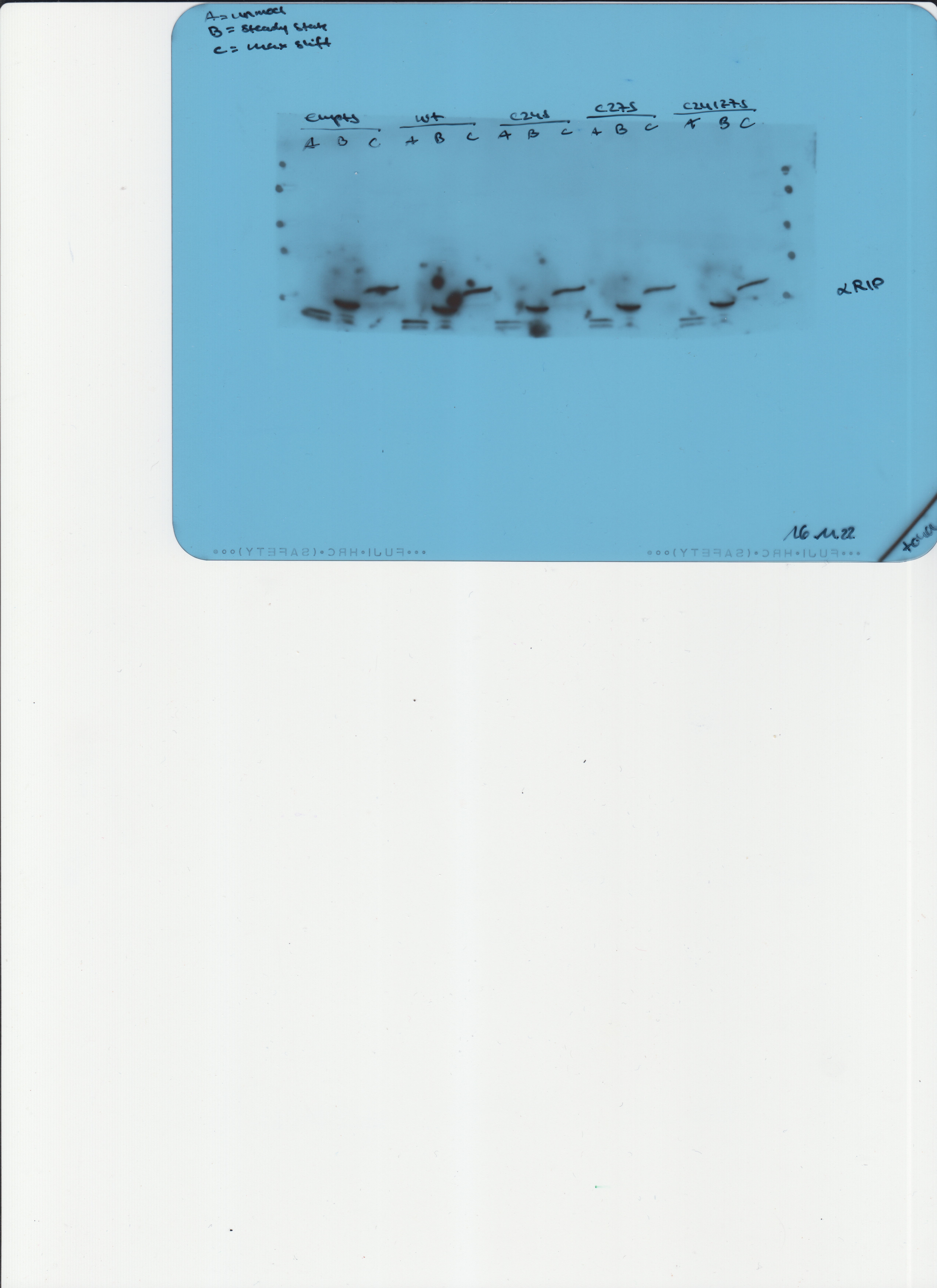

Supplement: Supplementary file 6 — Source data Fig. 5 [file 44319_2024_349_MOESM6_ESM.zip › Fig 5/A/shift wt delta Rip1.tiff]
