# Supplementary material for: Dbi1 is an oxidoreductase and an assembly chaperone for mitochondrial inner membrane proteins
Source: EMBO Rep. 2025 Jan 3;26(4):911–28. doi: 10.1038/s44319-024-00349-6 (PMC11850723; doi:10.1038/s44319-024-00349-6)
Supplement: Supplementary file 7 — Expanded View Figures [file 44319_2024_349_MOESM7_ESM.pdf]

Expanded View Figures

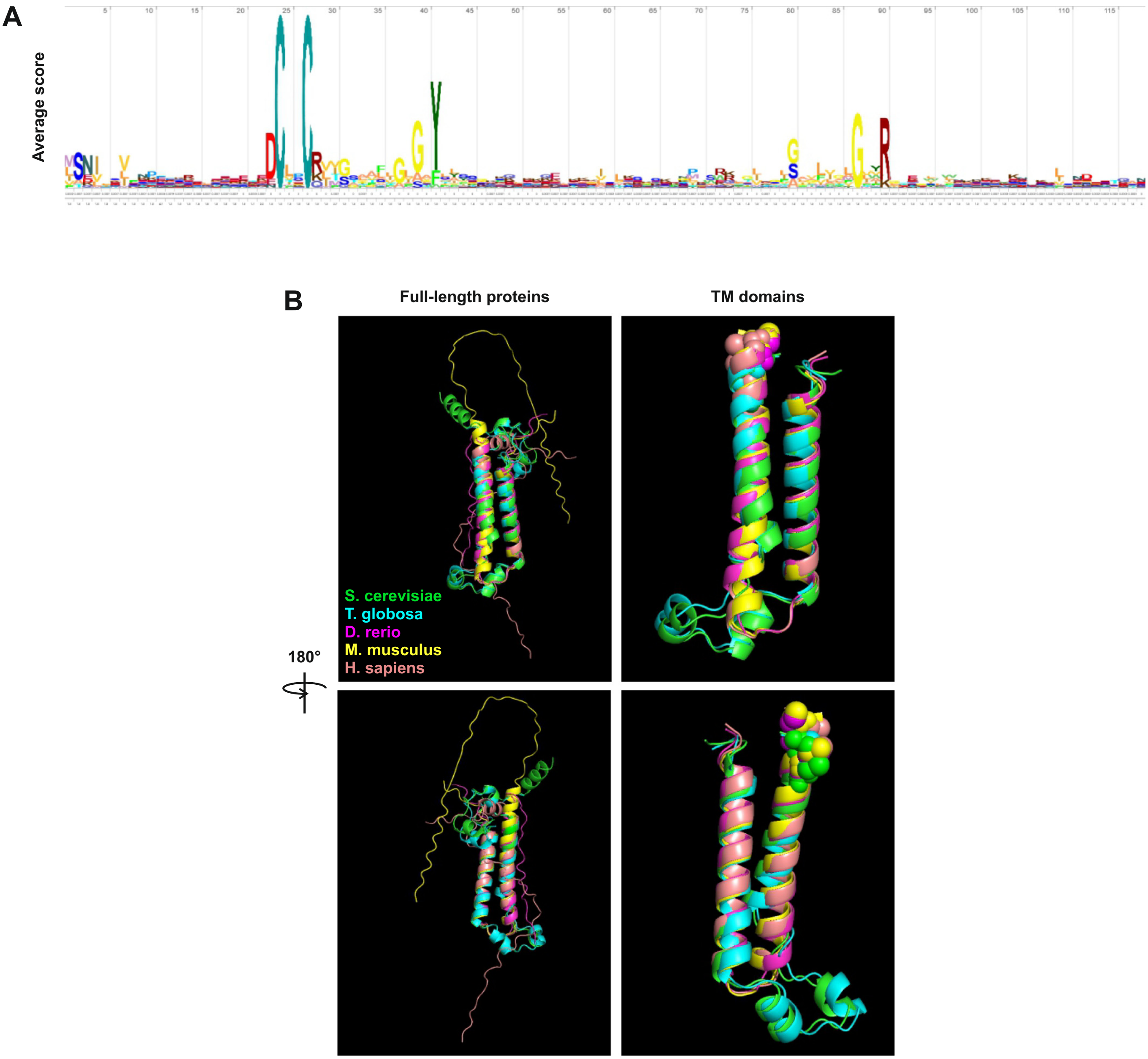

Figure EV1. Evolutionary conservation of Dbi1.

(**A**) *Saccharomyces cerevisiae* YDL157c protein sequence was used to search UniProtKB database using JACKHHMER online tool (https://www.ebi.ac.uk/Tools/hmmer/search/jackhmmer). After five iterations, 1136 proteins were identified, 1128 of which were eukaryotic. Screenshot of the obtained sequence conservation is shown. (**B**) AlphFold3 generated models of Dbi1 from *Saccharomyces cerevisiae* and its homologs from *Torulaspora globosa*, *Danio rerio*, *Mus musculus* and *Homo sapiens* were aligned using PyMOL. Cysteine residues are shown as spheres.

© The Author(s)

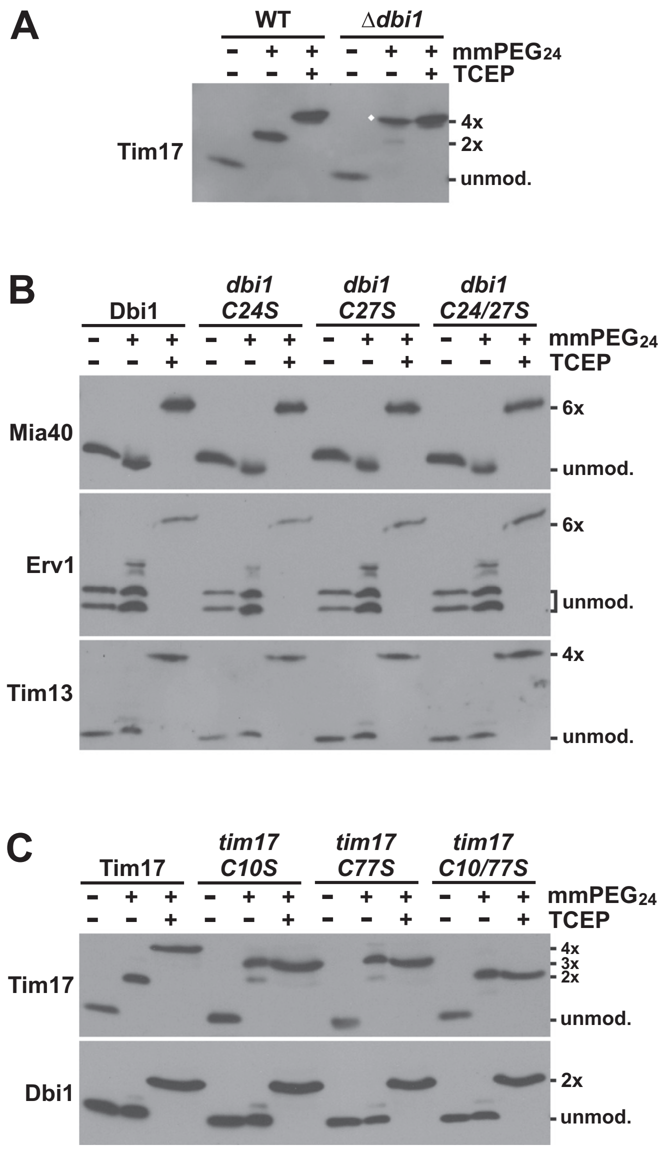

Figure EV2. Thiol modification in Dbi1- and Tim17 cysteine mutants.

(**A**) In vivo oxidation state of cysteine residues in cellular proteins was trapped by TCA-precipitation. Samples were solubilized in SDS-containing buffer and incubated with the free thiol reactive reagent methyl-polyethylene glycol-maleimide (mmPEG$_{24}$). One sample was fully reduced with TCEP at 96 °C prior to incubation with mmPEG$_{24}$. Samples were analyzed by SDS-PAGE and western blotting. White diamond highlights the fully reduced Tim17 in cells lacking Dbi1. (**B**) Isolated mitochondria containing indicated Dbi1 variants were solubilized in SDS-containing buffer and subsequently treated as in (**A**). (**C**) As in (**B**), with the difference that mitochondria containing Tim17 cysteine mutants were analyzed.

 © The Author(s)

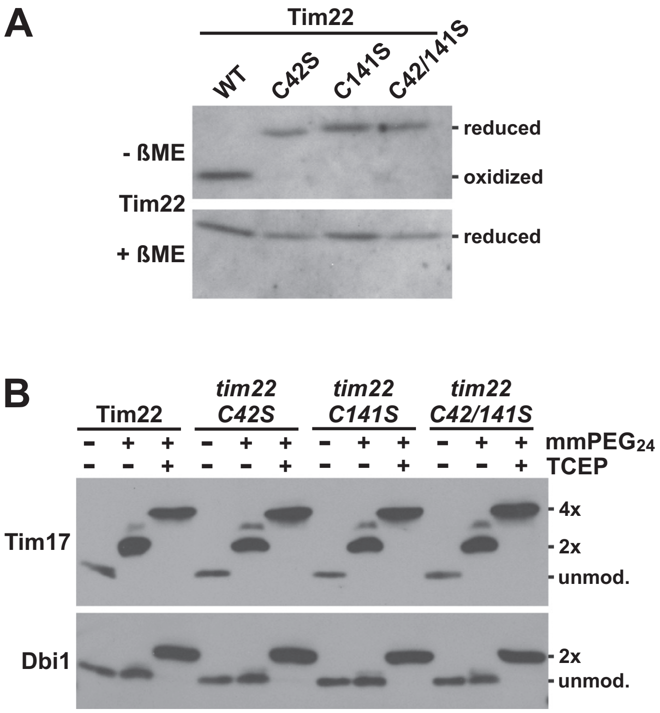

Figure EV3. Analysis of Tim22 cysteine mutants.

(**A**) Wild type and mitochondria containing indicated variants of Tim22 were dissolved in reducing (+ßME) or nonreducing (-ßME) Laemmli buffer and analyzed by SDS-PAGE and western blotting. (**B**) Isolated mitochondria containing indicated Tim22 variants were dissolved in SDS-containing buffer and incubated with methyl-polyethylene glycol-maleimide ($mmPEG_{24}$). One sample was fully reduced with TCEP at 96 °C prior to incubation with $mmPEG_{24}$. Samples were analyzed by SDS-PAGE and western blotting.

© The Author(s)

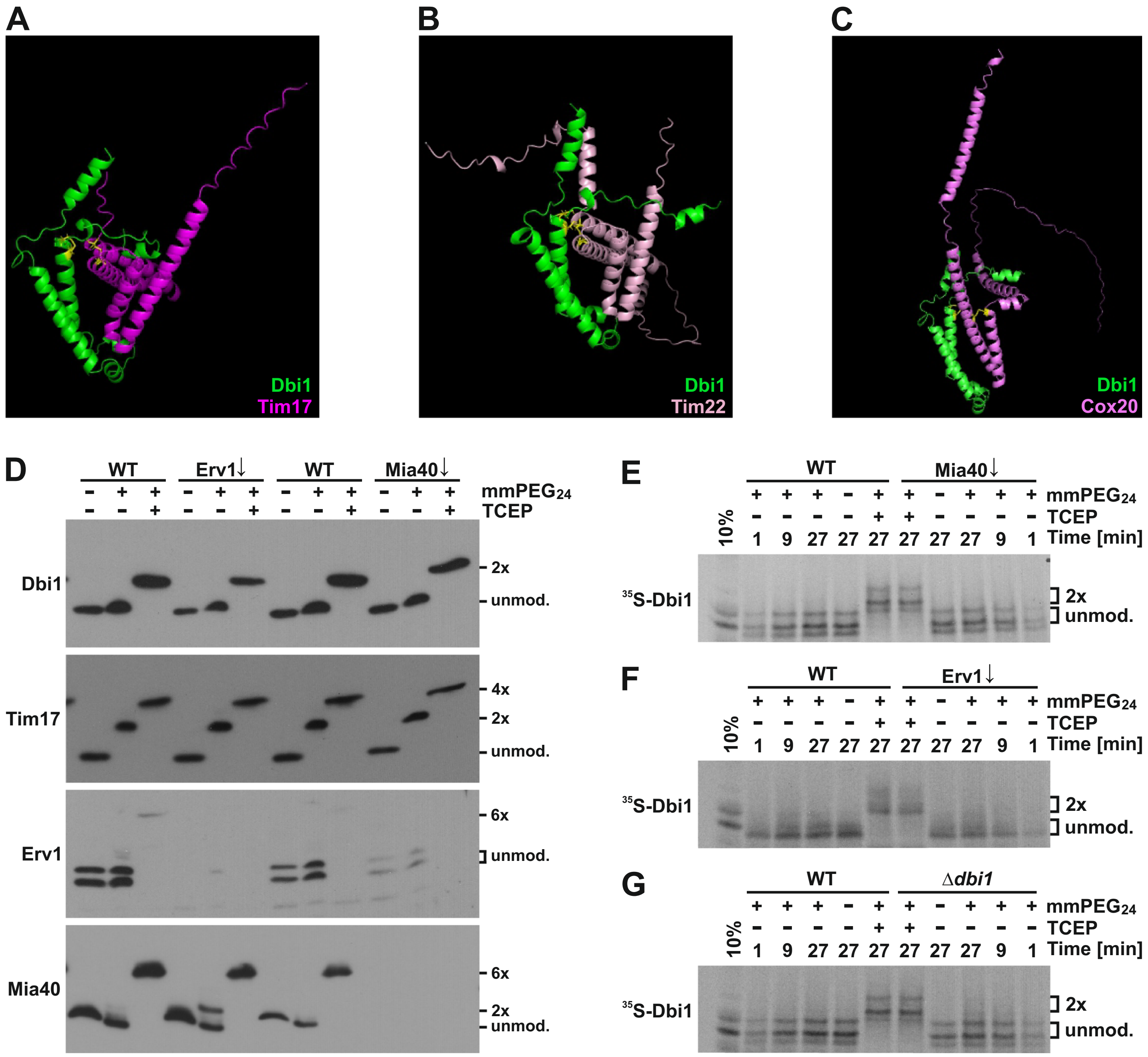

Figure EV4. Role of Dbi1 in oxidation and assembly of inner membrane proteins.

AlphaFold3 generated models of Dbi1 in complex with Tim17 (**A**), Tim22 (**B**), and Cox20 (**C**). Cysteine residues are shown as yellow sticks. (**D**) Isolated mitochondria were solubilized in SDS-containing buffer and incubated with $mmPEG_{24}$, where indicated. One sample was fully reduced with TCEP at 96 °C prior to labeling with $mmPEG_{24}$. Samples were analyzed by SDS-PAGE and western blotting using indicated antibodies. (**E–G**) ^{35}S-labeled Dbi1 was imported into isolated mitochondria. At indicated time points, samples were taken out, import was stopped and samples were treated with proteinase K to remove all nonimported material. Protease digestion was stopped by incubation with PMSF, mitochondria were reisolated, solubilized in SDS-containing buffer and incubated with $mmPEG_{24}$, where indicated. One sample was reduced with TCEP at 96 °C prior to incubation with $mmPEG_{24}$. Samples were analyzed by SDS-PAGE followed by autoradiography.

 © The Author(s)
